# Comparative transcriptomics of outbreak-associated *Salmonella* Heidelberg, Typhimurium, and monophasic Typhimurium reveals differential expression of stress tolerance, virulence, and biofilm-associated genes

**DOI:** 10.3389/fmicb.2026.1895105

**Published:** 2026-10-12

**Authors:** Chelsey A. Patch, Andrea J. Etter

**Affiliations:** Department of Nutrition and Food Sciences, The University of Vermont, Burlington, VT, United States

**Keywords:** foodborne outbreak, RNA-Seq, *Salmonella*, stress tolerance, transcriptomics, virulence

## Abstract

Non-typhoidal *Salmonella enterica* remains a leading cause of foodborne illness worldwide, with certain serovars more commonly implicated in outbreaks linked to specific food matrices. However, gene expression differences between distinct serovars remain poorly understood, which may limit the ability to reduce the overall outbreak and illness burden. This study presents an RNA-Seq evaluation of six outbreak-associated, stationary-phase *S. enterica* transcriptomes representing serovars *Salmonella* Heidelberg, *Salmonella* Typhimurium, and monophasic *Salmonella* Typhimurium (I 4,[5],12:i-). Analysis of differential gene expression (*p_adj_* < 0.05 and |log2FoldChange| > 1) revealed upregulation of genes associated with enhanced pathogenicity among all strains; however, *Salmonella* Heidelberg exhibited a transcriptional profile consistent with enhanced invasion, iron metabolism, and biofilm formation, while *Salmonella* Typhimurium and monophasic *Salmonella* Typhimurium showed transcriptional profiles consistent with stress response and survival. Transcriptome patterns were concordant with previous research evaluating phenotypic stress responses, in which *Salmonella* Heidelberg demonstrated prolonged survival at lethal temperatures and enhanced biofilm-forming capabilities. Overall, these findings suggest that outbreak-associated strains may employ distinct transcriptional strategies that could contribute to serovar-specific outbreak characteristics. This work supports risk assessment modeling to ultimately improve food safety interventions and reduce the public health burden of *S. enterica*.

## Introduction

Non-typhoidal *Salmonella enterica* is a leading cause of foodborne illness worldwide and is responsible for more than 1.3 million cases annually in the United States (Scallan Walter et al., 2025; WHO, 2015). *S. enterica* also accounts for the highest number of deaths among bacterial foodborne pathogens annually in the US, and it is a leading contributor to childhood morbidity and mortality from diarrheal disease globally (Scallan Walter et al., 2025; WHO, 2015). While not considered a ubiquitous organism, *S. enterica* is found naturally in a wide range of environments, including surface water (Liu et al., 2018), poultry (CDC, 2021; Clothier et al., 2018; Craven et al., 2000; Karabozhilova et al., 2012; Keerthirathne et al., 2022; Nichols et al., 2021; Parzygnat et al., 2024; Patch et al., 2025; Stapleton et al., 2024; Tizard, 2004), wild birds (CDC, 2021; Craven et al., 2000; Tizard, 2004), mammals (Kawakami et al., 2019; Nichols et al., 2022; Roug et al., 2013), and reptiles (Marus et al., 2019). Consequently, *S. enterica* poses a contamination risk in many food products and has been associated with outbreaks involving foods including nuts and nut butters (Cavallaro et al., 2011; CDC, 2009; Pereira et al., 2020), raw produce (Allard et al., 2013; Campbell et al., 2001; CDC, 2015b; Hanning et al., 2009; Jackson et al., 2013), and spices (Bakker et al., 2011), as well as more traditional animal-derived products including meat, eggs, and poultry (Gieraltowski et al., 2016; Kawakami et al., 2019; Lanier et al., 2018).

Of the more than 2,600 genetically distinct *Salmonella* serovars, only a subset is commonly implicated in illnesses and outbreaks. The top five isolated from both human clinical samples and food sources include *Salmonella* Enteritidis, *Salmonella* Typhimurium, and monophasic *Salmonella* Typhimurium (I 4,[5],12:i-), while serovars such as *Salmonella* Heidelberg are often isolated from poultry, and *Salmonella* Senftenberg from turkey (Han et al., 2024). Furthermore, between 1998 and 2021, serovars *Salmonella* *Typhimurium*, monophasic *Salmonella* Typhimurium, and *Salmonella* Heidelberg were among the top six causative serovars of outbreaks, with the top three food vehicle categories being chicken, eggs, and pork (Buyrukoglu et al., 2024). Thus, certain *S. enterica* serovars and food products are disproportionately responsible for the overall disease burden (Buyrukoglu et al., 2024). To reduce infections attributable to *Salmonella*, the US Department of Health and Human Services established the Healthy People goals; however, they were not met in 2010 or 2020 (Davis, 2000; USDHHS, 2020). As the USDA has proposed regulating *Salmonella* in poultry using a serovar-specific approach for mitigation, understanding attributes unique to each serovar is critical (Proposed Salmonella framework for raw poultry products, RIN 0583-AD96 C.F.R. 2024 Federal Register, 2024).

The ability of many *S. enterica* serovars to develop tolerance to various stresses contributes to both persistence in processing environments and to human illness (Dawoud et al., 2017; Lanier et al., 2018). To persist in the food processing environment, *S. enterica* must survive heat treatment, food contact antimicrobials, and sanitizers (commonly acid-based or chlorine-based); likewise, to establish infection in the host, *S. enterica* must overcome various stressors including low pH in the acidic environment of the stomach and oxidative stress in macrophages (Guillén et al., 2021). Furthermore, *S. enterica* is able to rapidly reprogram metabolic pathways to enhance pathogenesis in virulence- and growth-focused modes, or in stress-response modes, emphasizing its diversity and adaptability (Guillén et al., 2021). This makes *S. enterica* a particularly difficult pathogen to combat, but understanding serovar variations and genomic adaptations can drive both pre-harvest and processing remediation and intervention strategies.

Numerous studies have assessed genomic differences among a variety of *S. enterica* serovars, searching for genetic determinants that contribute to virulence and pathogenicity. However, these comparative genomic analyses have largely failed to identify specific genes that definitively account for virulence differences among NTS serovars. Furthermore, phenotypic divergence in virulence, stress tolerance, and overall pathogenicity may be driven primarily by differential gene expression rather than differences in gene content alone. Nonetheless, comparative data on gene expression patterns across several distinct serovars of outbreak-associated *S. enterica* remain limited.

We recently conducted a study of stress tolerance in 43 food isolates across five serovars from five outbreaks to evaluate overall stress tolerance, and found heat tolerance at lethal temperatures (56 °C), sanitizer tolerance (sodium hypochlorite and peracetic acid), antimicrobial resistance (AMR), heavy metal tolerance, and enhanced biofilm-forming abilities among certain isolates (Martin et al., 2026). Most notably, *Salmonella* serovar Heidelberg isolates from the 2013–2014 chicken and 2011 kosher broiled chicken liver outbreaks showed high levels of AMR, peracetic acid (PAA) tolerance, survival at lethal temperatures, and strong biofilm-forming abilities across multiple conditions; monophasic *Salmonella* Typhimurium isolates from the 2015 roast pork outbreak displayed enhanced heavy metal tolerance, survival at lethal temperatures, and the lowest initial heat inactivation; and *Salmonella* Typhimurium isolates from the 2008–2009 peanut outbreak displayed high chlorine-based sanitizer (NaOCl) tolerance and strong biofilm-forming capacity under nutrient-depletion conditions (Martin et al., 2026). These phenotypic characteristics may have contributed to the scope and severity of their respective outbreaks. Characterizing stationary-phase gene expression can improve our understanding of complex transcriptomic differences across serovars and outbreaks, aiding the refinement of risk modeling, food processing control points, and therapeutic targets.

The objectives of this study were to conduct RNA sequencing on six of these outbreak-associated isolates to: (i) compare the transcriptomic profiles with phenotypic stress tolerance data previously obtained, and (ii) analyze transcriptomic differences among strains that may contribute to their overall virulence and pathogenicity.

## Materials and methods

### Selection of bacterial strains

Isolates were sourced from the USDA Culture Collection, the New York State Food Laboratory, and the FDA. The selected isolates included two monophasic *Salmonella* Typhimurium (I 4,[5],12:i:-) strains (FML-M2-0012 and FML-M2-0013) from the 2015 roast pork outbreak, which resulted in 188 illnesses and a 16% hospitalization rate (CDC, 2015a); two *Salmonella* Heidelberg isolates from the 2011 kosher broiled chicken liver outbreak (FML-M2-0030 and FML-M2-0031), which resulted in 190 illnesses and a 19% hospitalization rate (CDC, 2012); one *Salmonella* Typhimurium isolate from the 2008–2009 peanut butter outbreak (FML-M2-0047), which resulted in 714 illnesses, a 24% hospitalization rate, and nine deaths (CDC, 2009); and one *Salmonella* Heidelberg isolate from the 2013 to 2014 chicken outbreak (FML-M2-0001), which resulted in 634 illnesses, with a 38% hospitalization rate and 15% of patients developing bacteremia (CDC, 2014). Isolate information, along with key genomic findings from Martin et al., 2026, is shown in Table 1.

**Table 1:** Characteristics of isolates used for RNA-Seq.

Characteristic	FML-M2-0001	FML-M2-0030	FML-M2-0031	FML-M2-0012	FML-M2-0013	FML-M2-0047
Isolate	1	4	5	2	3	6
Serovar	Heidelberg	Heidelberg	Heidelberg	Monophasic Typhimurium	Monophasic Typhimurium	Typhimurium
Outbreak	Chicken, 2013–2014	Kosher broiled chicken liver, 2011	Kosher broiled chicken liver, 2011	Roast pork, 2015	Roast pork, 2015	Peanut butter, 2008–2009
Outbreak Characteristics	634 illnesses, 38% hospitalization rate, 15% blood infection rate	190 illnesses, 19% hospitalization rate	190 illnesses, 19% hospitalization rate	188 illnesses, 16% hospitalization rate	188 illnesses, 16% hospitalization rate	714 illnesses, 24% hospitalization rate, 9 deaths
Source	Chicken tenderloin	Fresh chopped chicken liver	Broiled chicken liver	Market Swine	Market Swine	Peanut butter paste
Obtained From	USDA Culture Collection	NY State Food Laboratory	NY State Food Laboratory	USDA Culture Collection	USDA Culture Collection	FDA
Biosample Accession	SAMN02709124	SAMN06672001	SAMN06672000	SAMN04125694	SAMN04125695	SAMN02678704
Isolate Name Alias	B-65037, B-59140, FSIS-FY14-1	11B09799A-1, CFSAN062518	11B09801A-1, CFSAN062519	B-65412, FSIS1503789	B-65413, FSIS1503790	SAL 9672
cgMLST Type	14,616	8,100	8,100	10,418	8,618	17,662
Heat Shock (last time point with live cells at 56 °C)	45a	45	60+	60+	15	45
Genotypic AMR	Aminoglycosides, bleomycin, fosfomycin, sulfonamides, tetracycline	Fosfomycin	Fosfomycin	Aminoglycosides, beta-lactams, sulfonamides, tetracycline	Aminoglycosides, beta-lactams, sulfonamides, tetracycline	None
Heavy Metal Tolerance	Tellurium, copper, silver, gold, arsenic	Mercury, gold, arsenic	Gold, arsenic	Mercury, copper, silver, gold, arsenic	Mercury, copper, silver, gold, arsenic	Gold, arsenic
NaOCl MIC (ppm in 1/20X TSB)	100	150	>200	50	50	>200
Biofilm: Condition tested with the highest biomass (OD_600_)	Nutrient abundance, room temp	Nutrient depletion, room temp	Nutrient depletion, room temp	Nutrient abundance, room temp	Nutrient abundance, room temp	Nutrient depletion, room temp

^a^Heat shock data from a previously published study (Etter et al., 2019).

### Isolate growth conditions

Isolates were recovered from working stock solutions by streaking 10 microliters onto fresh tryptic soy agar (Becton Dickinson; Franklin Lakes, NJ) plates using a four-quadrant streak method and incubated at 37 °C for 24 h. A single colony from each plate was aseptically transferred to 10 mL of fresh tryptic soy broth (TSB) and incubated at 37 °C for 16 h with shaking at 200 rpm. After incubation, each culture was serially diluted (five 10-fold dilutions) to reach approximately 4 log_10_ CFU/mL, and the diluted cultures were reincubated at 37 °C for 8 h with shaking (200 rpm).

### RNA extraction, library preparation, and RNA-sequencing

Total RNA was extracted from early-stationary-phase cultures grown at 37 °C in TSB using the PureLink™ RNA Mini Kit according to the manufacturer’s instructions (Invitrogen, Waltham, MA). Library preparation was performed using the Zymo-Seq RiboFree Total RNA Library Kit (Zymo Research, Irvine, CA). RiboDetector was used to determine rRNA depletion efficiency (Deng et al., 2022), and revealed that greater than 90% + depletion was achieved with the Zymo-Seq RiboFree kit. Libraries prepared with Illumina adapters were converted to Singular adapters using the Singular Genomics (SG) Library Compatibility Kit (Part # 700141; Singular Genomics, San Diego, CA) and sparQ HiFi PCR Master Mix (Quantabio, Beverly, MA). The Illumina-to-Singular converted libraries were sequenced on two F3 flow cells as 2 × 150 bp paired-end reads using the SG G4 sequencing system (SG, San Diego, CA). SG primers (Part # 700142) for the index reads were spiked into a 300-cycle reagent kit (Part #700129) (SG, San Diego, CA).

### Data processing

Quality assessment was performed for fastq files using FastQC v0.12.1 (Andrews, 2010) and MultiQC v1.0 (Ewels et al., 2016). Reads were trimmed to remove Illumina adapters and low-quality bases with Phred scores < 20 using Trim Galore v0.6.10^1^ and Cutadapt (Martin, 2011), followed by a secondary quality assessment using FastQC v0.12.1 and MultiQC v1.0. Reads were aligned to a concatenated reference genome (GCF_000020705.1: Heidelberg SL476, GCF_000170255.1: monophasic Typhimurium CVM23701, and GCF_000006945.2: Typhimurium LT2) using minimap2 v2.28 (Li, 2018) to ensure serovar-specific alignment. Alignment quality was assessed using samtools stats, prior to read counting with HTSeq v2.0.5 (Putri et al., 2022). Genome quality metrics are shown in Supplementary Table S1. Differential gene expression was analyzed using DESeq2 v1.38.3 (Love et al., 2014) in R v4.2.1 (R Core Team, 2022). Genes with a baseMean read count of 0 across all three biological replicates per isolate were filtered out. Genes with a *p_adj_* value of <0.05 and |log2FoldChange| > 1 were considered differentially expressed genes (DEGs). DEGs were annotated using Gene Ontology (GO) annotations, Basic Local Alignment Search Tool (BLAST), and manual curation. Pathway analysis was performed using the PANTHER classification system (version 19.0^2^) and the overrepresentation test (*Salmonella* Typhimurium as reference), annotated by GO biological process terms.

### Data availability

Raw sequencing reads are available in the NCBI Sequence Read Archive (SRA) under BioProject PRJNA1241831, and read count files will be uploaded to NCBI-GEO and released upon publication of this manuscript.

## Results

Our goal was to investigate differences in stationary-phase gene expression profiles of six outbreak-associated S. enterica strains from multiple serovars with previously characterized phenotypic stress tolerance (Martin et al., 2026) using RNA-sequencing and subsequent transcriptomic analysis. The sequence quality of the isolates was high, with a mean index base quality (Phred) of 34.07, a mean raw reads per sample of 81,993,823 (77,682,559 to 89,374,489), and a mean alignment rate of 97.3% (95.6–98.3). Three pairwise comparisons were performed using DESeq2 to evaluate differences between serovars: comparison 1 was between monophasic *Salmonella* Typhimurium (isolates FML-M2-0012 and FML-M2-0013) and *Salmonella* Heidelberg (isolates FML-M2-0001, FML-M2-0030, and FML-M2-0031, the reference serovar), comparison 2 was between *Salmonella* Typhimurium (isolate FML-M2-0047) and *Salmonella* Heidelberg (isolates FML-M2-0001, FML-M2-0030, and FML-M2-0031, the reference serovar), and comparison 3 was between *Salmonella* Typhimurium (isolate FML-M2-0047) and monophasic *Salmonella* Typhimurium (isolates FML-M2-0012 and FML-M2-0013, the reference serovar). While *Salmonella* Heidelberg and *Salmonella* monophasic Typhimurium biological replicates clustered strongly by serovar., *Salmonella* Typhimurium replicates did not cluster closely together in a principal component analysis (PCA) or hierarchical dendrogram (Figures 1A,B).

**Figure 1:**
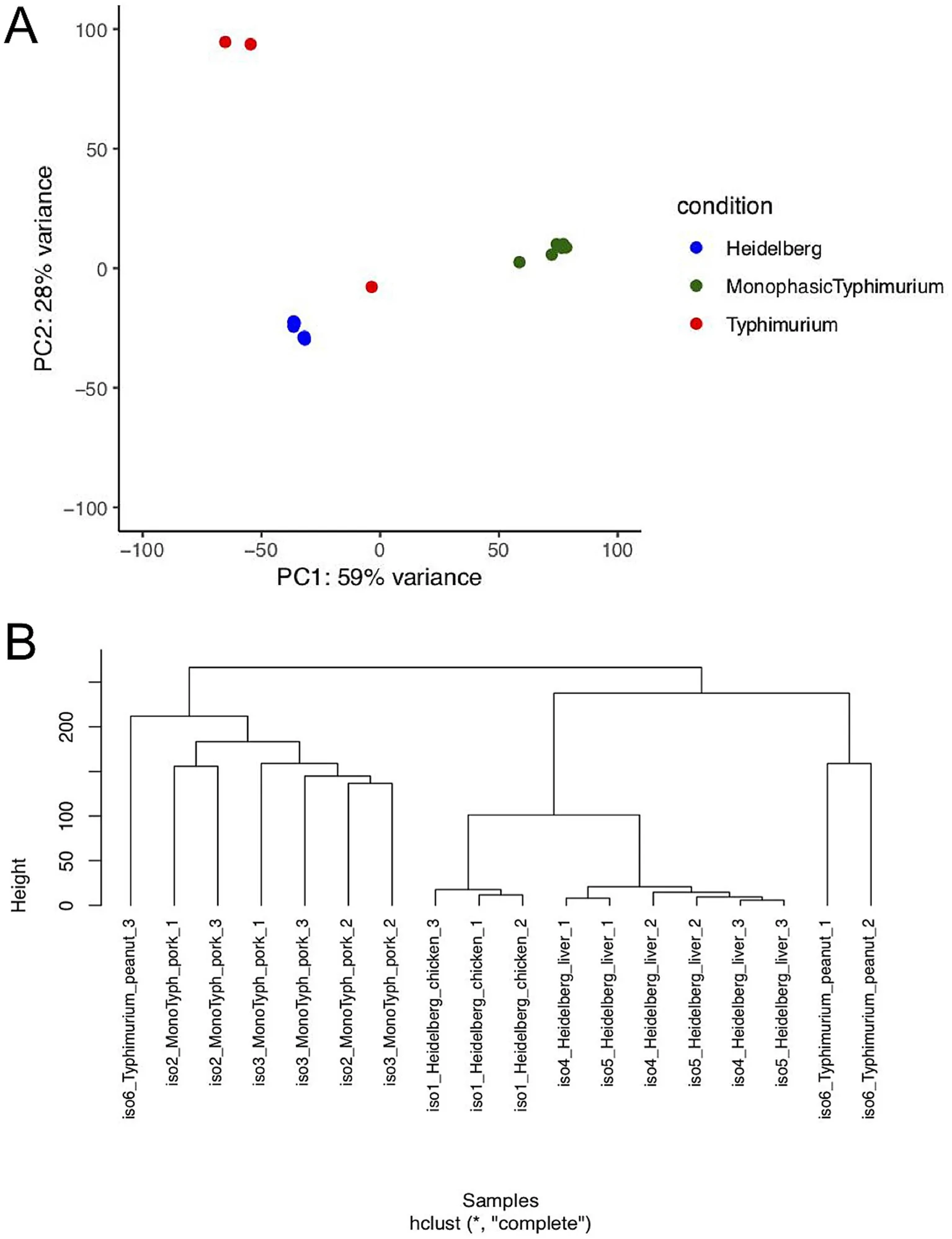
**(A)** Principal component analysis (PCA) plot and **(B)** Dendrogram showing overall relatedness by sample; *Salmonella* Typhimurium isolate 6, replicate 3 (FML-M2-0047; iso6_Typhimurium_peanut_3) grouped more closely to monophasic *Salmonella* Typhimurium isolates than other *Salmonella* Typhimurium replicates.

The three pairwise comparisons yielded a substantial number of DEGs, as shown in Figure 2. The monophasic variant of Typhimurium and classic *Salmonella* Typhimurium isolates had highly similar stationary-phase gene expression profiles; only 17 genes were differentially expressed between the two *Salmonella* Typhimurium serovar variants. In contrast, *Salmonella* Heidelberg had a high number of DEGs when compared with *Salmonella* Typhimurium (*n* = 410) and monophasic *Salmonella* Typhimurium (*n* = 343), with 123 shared DEGs identified in *Salmonella* Heidelberg relative to both *Salmonella* Typhimurium and monophasic *Salmonella* Typhimurium.

**Figure 2:**
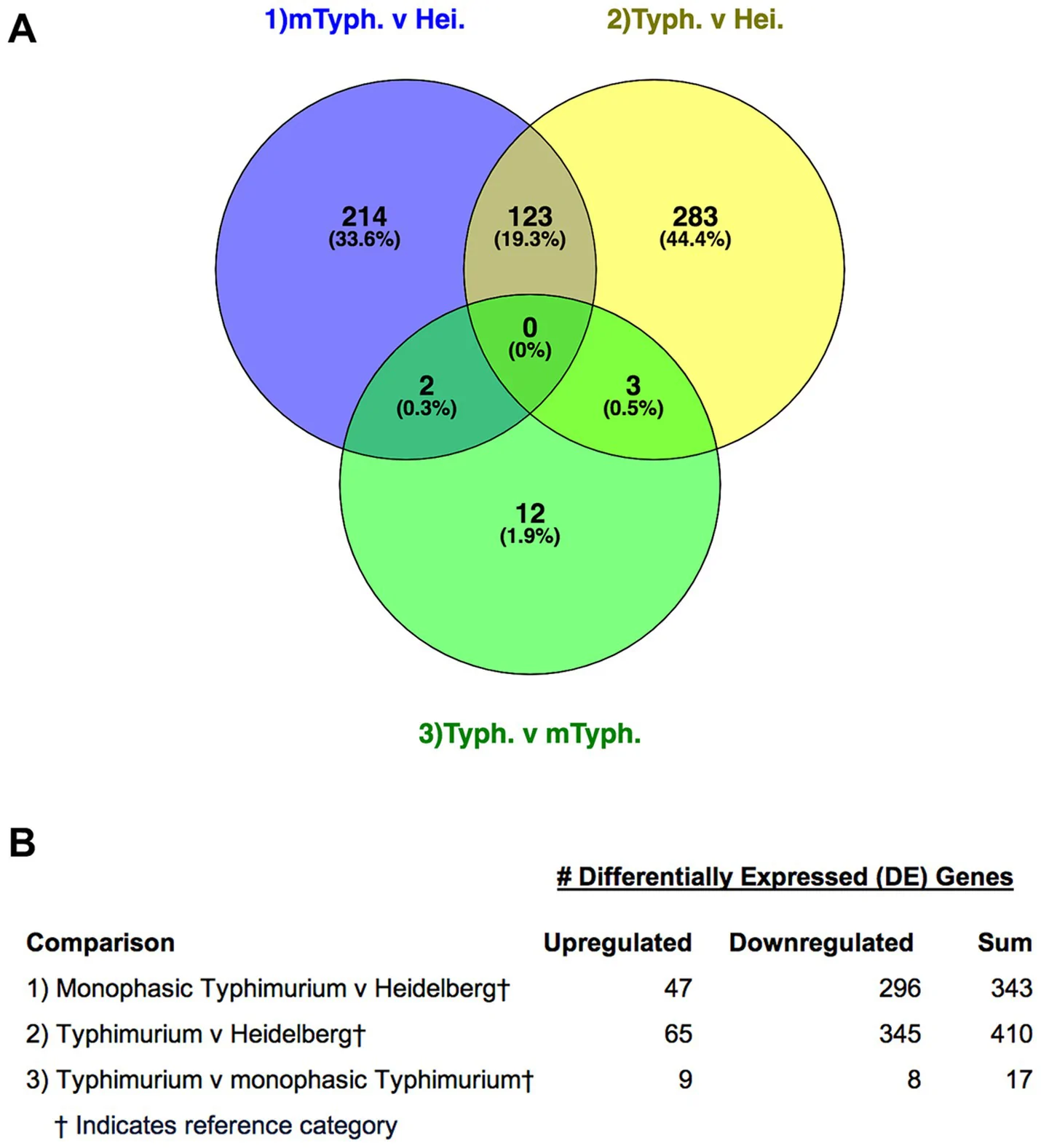
Venn diagram displaying differentially expressed (DE) genes **(A)**; Chart detailing the number of upregulated, downregulated, and total DEGs by pairwise comparison **(B)**.

### Pathway analyses

A pathway enrichment analysis was completed for all annotated DEGs to identify the functional molecular classifications that were most prevalent. When comparing monophasic *Salmonella* Typhimurium and *Salmonella* Heidelberg isolates (Supplementary Figure S1A), most DEGs were involved in catalytic activity (57.1%), followed by binding (21.4%), and transporter activity (12.5%), with certain genes matching multiple functional categories. However, nearly half (*n* = 179/343) of the DEGs were assigned to no PANTHER/GO pathway term (“unclassified”) and were consequently excluded from this analysis. Similarly, when comparing *Salmonella* Typhimurium and *Salmonella* Heidelberg isolates (Supplementary Figure S1B), DEGs were predominantly involved in catalytic activity (61.1%), binding (18.5%), and transporter activity (11%); 209/343 DEGs were unclassified and excluded. Finally, comparing monophasic *Salmonella* Typhimurium with *Salmonella* Typhimurium (Supplementary Figure S1C), 5/10 classified hits were associated with catalytic activity, followed by binding (37.5%) and translation regulator activity (12.5%); 7/17 DEGs were unclassified and excluded.

A PANTHER overrepresentation test was performed on all DEGs, examining predicted and observed gene prevalence via Fisher’s exact test to identify functional pathways significantly enriched beyond random expectation. When compared with monophasic *Salmonella* Typhimurium, *Salmonella* Heidelberg upregulated pathways (FDR-adjusted *p* < 0.05) included biological and cellular processes. When compared with *Salmonella* Typhimurium, upregulated pathways in *Salmonella* Heidelberg isolates included protoporphyrinogen IX biosynthetic and metabolic processes, menaquinone biosynthetic and metabolic processes, alcohol metabolic processes, organic hydroxy compound metabolic processes, small molecule metabolic processes, biosynthetic processes, metabolic processes, primary metabolic processes, cellular processes, and biological processes (detailed GO process terms, fold-change values, and *p*-values are provided in Supplementary Table S2). There were no statistically significant pathway enrichments detected between monophasic *Salmonella* Typhimurium and *Salmonella* Typhimurium isolates.

### DEG overview

Annotated DEGs were assessed for their relevance to virulence, stress tolerance, biofilm formation, and infection potential. Overall, 37.9% (*n* = 130/343) of DEGs from the monophasic *Salmonella* Typhimurium vs. *Salmonella* Heidelberg comparison, 35.4% (*n* = 145/410) of genes from the *Salmonella* Typhimurium vs. *Salmonella* Heidelberg comparison, and 35.3% (*n* = 6/17) of DEGs from the *Salmonella* Typhimurium vs. monophasic *Salmonella* Typhimurium comparison were selected for further functional characterization. These genes were broadly categorized by their association with antimicrobial resistance (AMR), biofilm formation, DNA repair, the heat shock response, heavy metal tolerance, general stress response, and virulence, with several genes belonging to multiple classifications.

A heatmap of log-normalized counts for shared genes across all three serovars is shown in Figure 3. The majority of genes (*n* = 43/47) were expressed at higher levels in *Salmonella* Heidelberg isolates compared with the other two serovars. However, genes associated with biofilm formation, heavy metal tolerance, stress response, and virulence were expressed at higher levels in monophasic *Salmonella* Typhimurium, *Salmonella* Typhimurium, or both serovars. Notably, *wzb*, encoding a low-molecular-weight protein–tyrosine phosphatase associated with colonic acid biosynthesis and biofilm formation, was expressed at higher levels in both *Salmonella* Typhimurium variants. Likewise, *cpxR*, encoding a transcriptional regulatory protein involved in the envelope stress response, and *ssrA*, located in *Salmonella* pathogenicity island (SPI) 2 associated with type 3 secretion systems (T3SS), were both expressed at significantly lower levels in *Salmonella* Heidelberg isolates. The *arsR* gene, associated with arsenic resistance, was expressed at high levels in the monophasic *Salmonella* Typhimurium isolates when compared with the other two serovars. Overall, normalized gene counts for monophasic *Salmonella* Typhimurium and *Salmonella* Typhimurium samples were highly similar. As expected, annotated DEGs between the two *Salmonella* Typhimurium variants (*n* = 6) included the AMR gene *tetR(B)*, the stress response gene *ymdB*, and the virulence gene *aroA*, which were all downregulated in the monophasic variant. Upregulated genes included the virulence genes *pepN*, *yeiP*, and flagellar repressor *fljA*, the latter of which is explained by the structural flagellar differences between the two variants.

**Figure 3:**
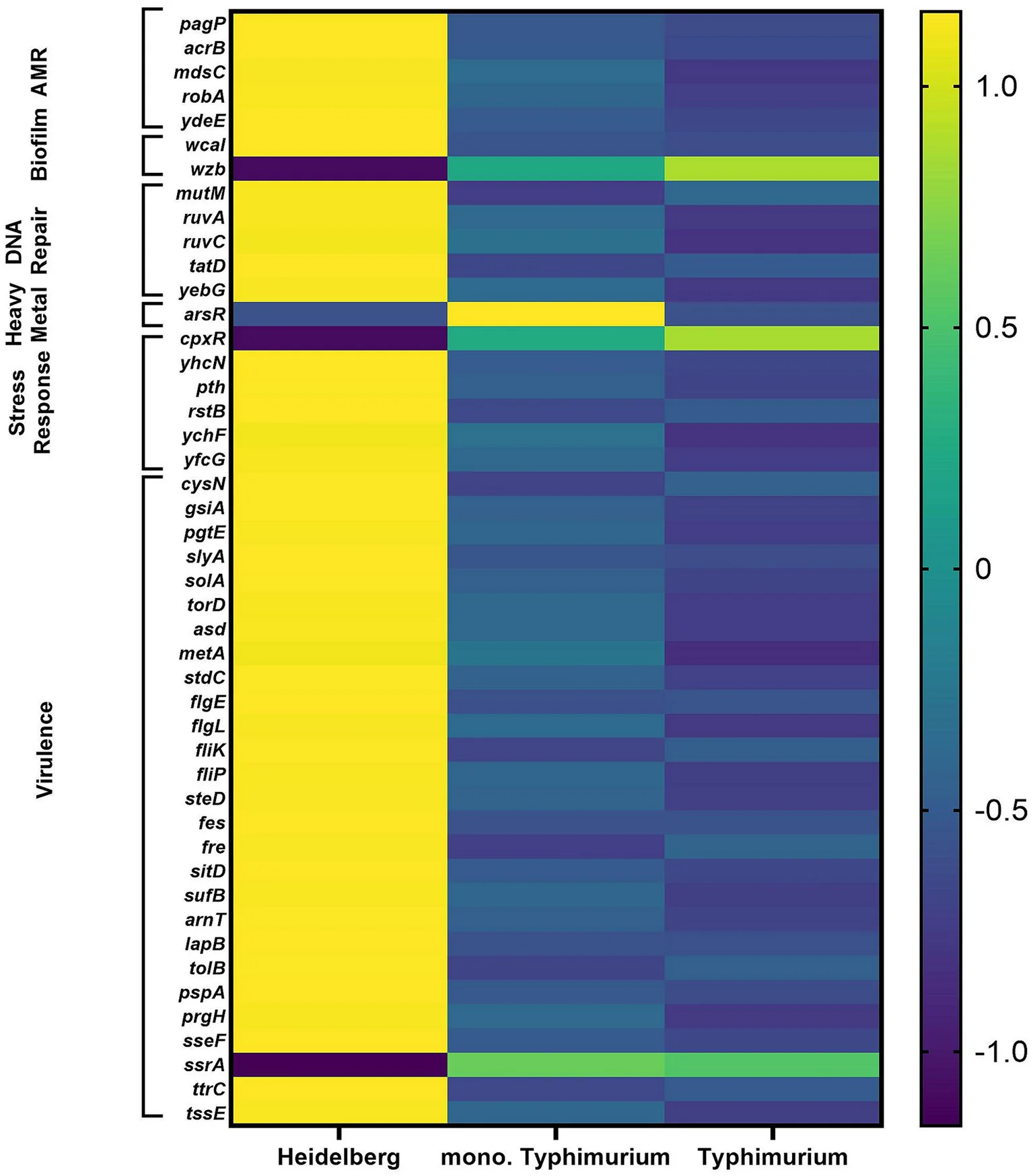
Heatmap showing upregulation and downregulation of DEGs by serovar., using *Z*-Score enrichment based on normalized counts.

The top 30 selected DEGs for the monophasic *Salmonella* Typhimurium vs. *Salmonella* Heidelberg and S. typhimurium vs. *S.* Heidelberg comparisons are shown in Figure 4. DEG expression patterns for factors associated with Salmonella survival were evaluated: AMR (Figure 5A), biofilm formation (Figure 5B), quorum sensing (Figure 5C), and heat shock, DNA repair, and heavy metal tolerance (Figure 5D). Furthermore, genes associated with stress response (Figure 6) and virulence (Figures 7, 8) constituted the majority of DEG.

**Figure 4:**
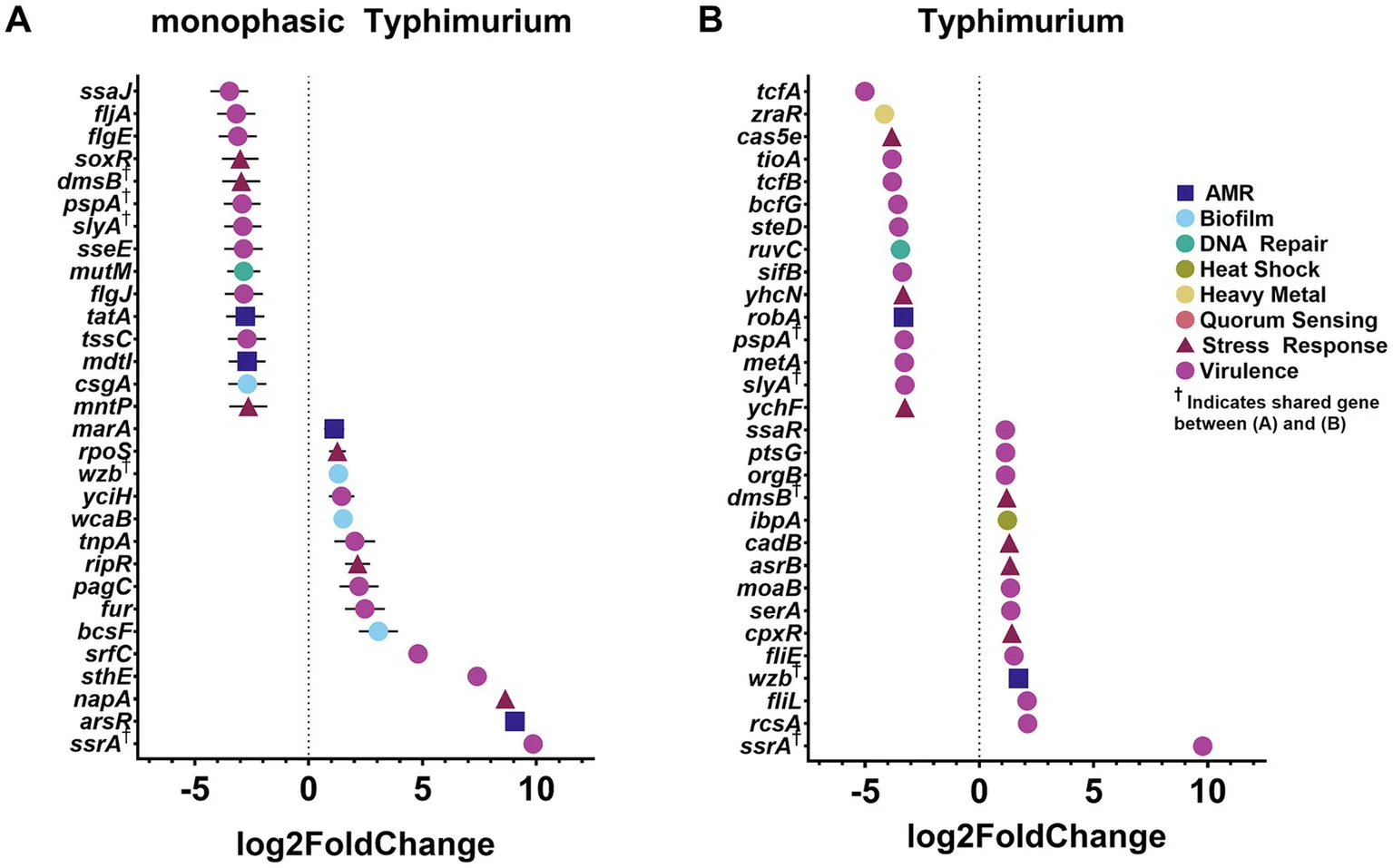
Top 30 selected DEGs among monophasic *Salmonella* Typhimurium and *Salmonella* Typhimurium isolates when compared to *Salmonella* Heidelberg, the reference category. DEGs were highly similar across both comparisons.

**Figure 5:**
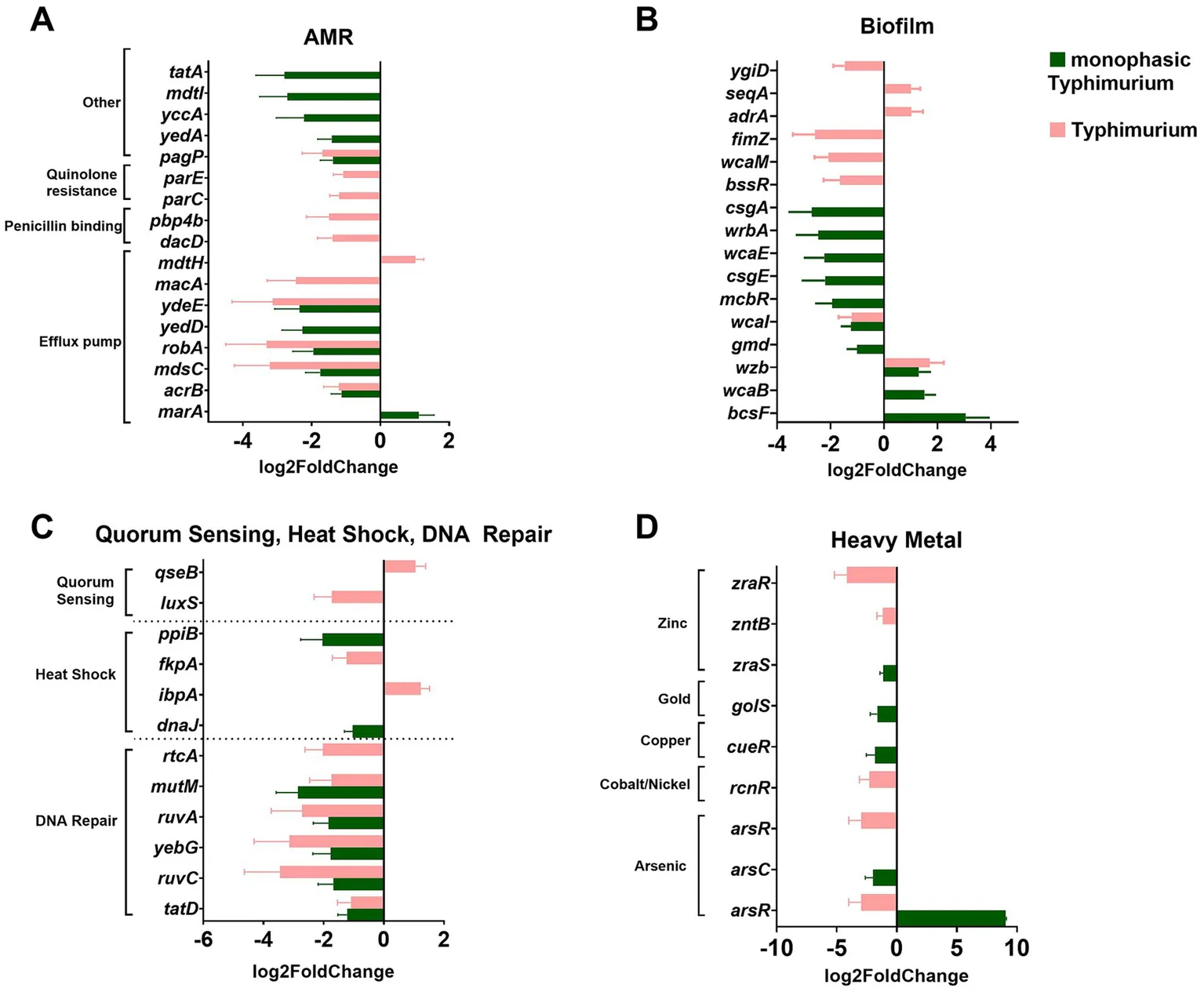
All selected DEGs by primary association in relation to overall infection potential and pathogenicity among monophasic *Salmonella* Typhimurium and *Salmonella* Typhimurium isolates when compared to *Salmonella* Heidelberg isolates, the reference category, including genes associated with antimicrobial resistance, biofilm formation, quorum sensing, heat shock, DNA repair, and heavy metal tolerance.

**Figure 6:**
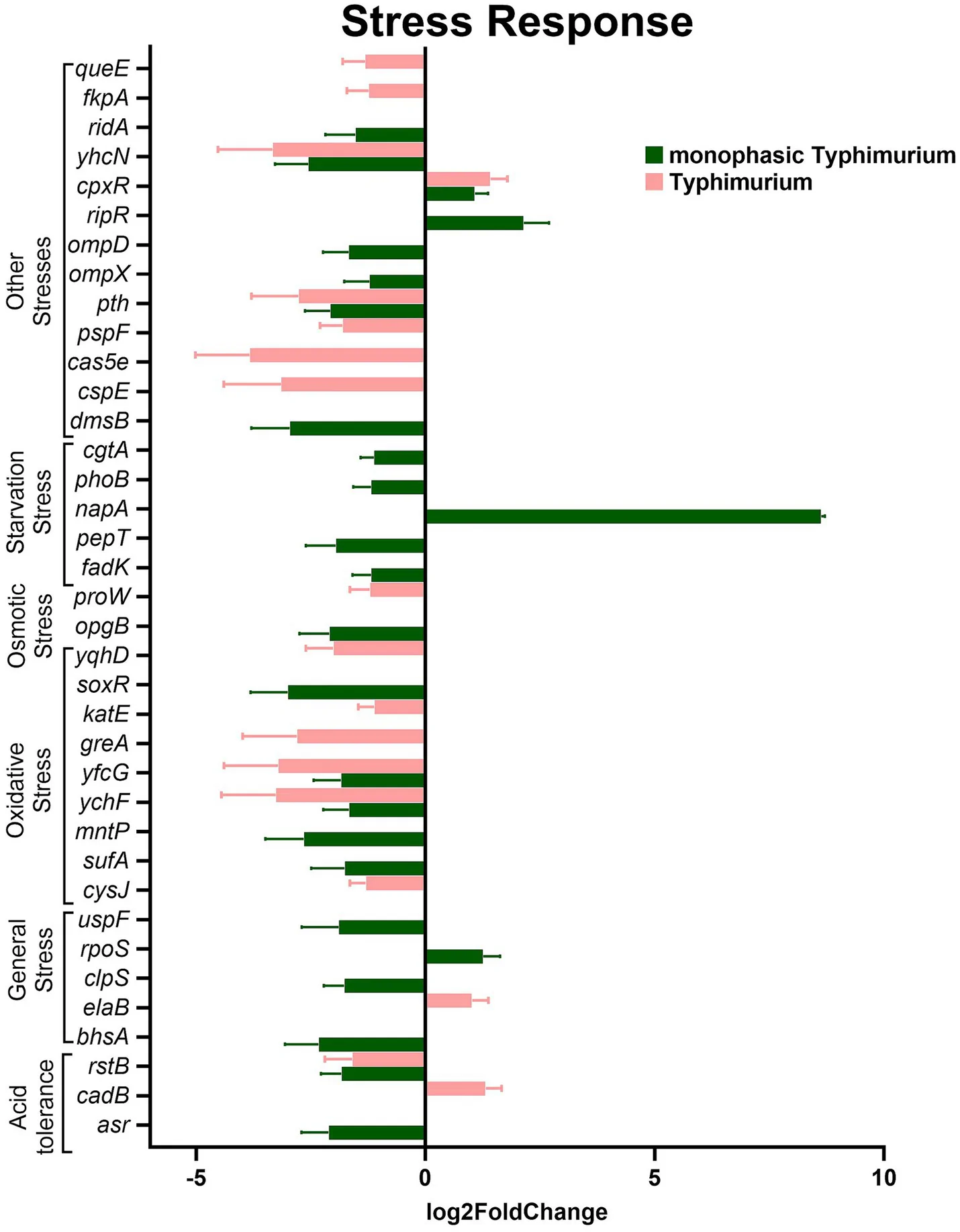
All stress-response-associated selected DEGs by primary association in relation to overall infection potential and pathogenicity among monophasic *Salmonella* Typhimurium (green) and *Salmonella* Typhimurium (pink) isolates when compared to *Salmonella* Heidelberg isolates, the reference category.

**Figure 7:**
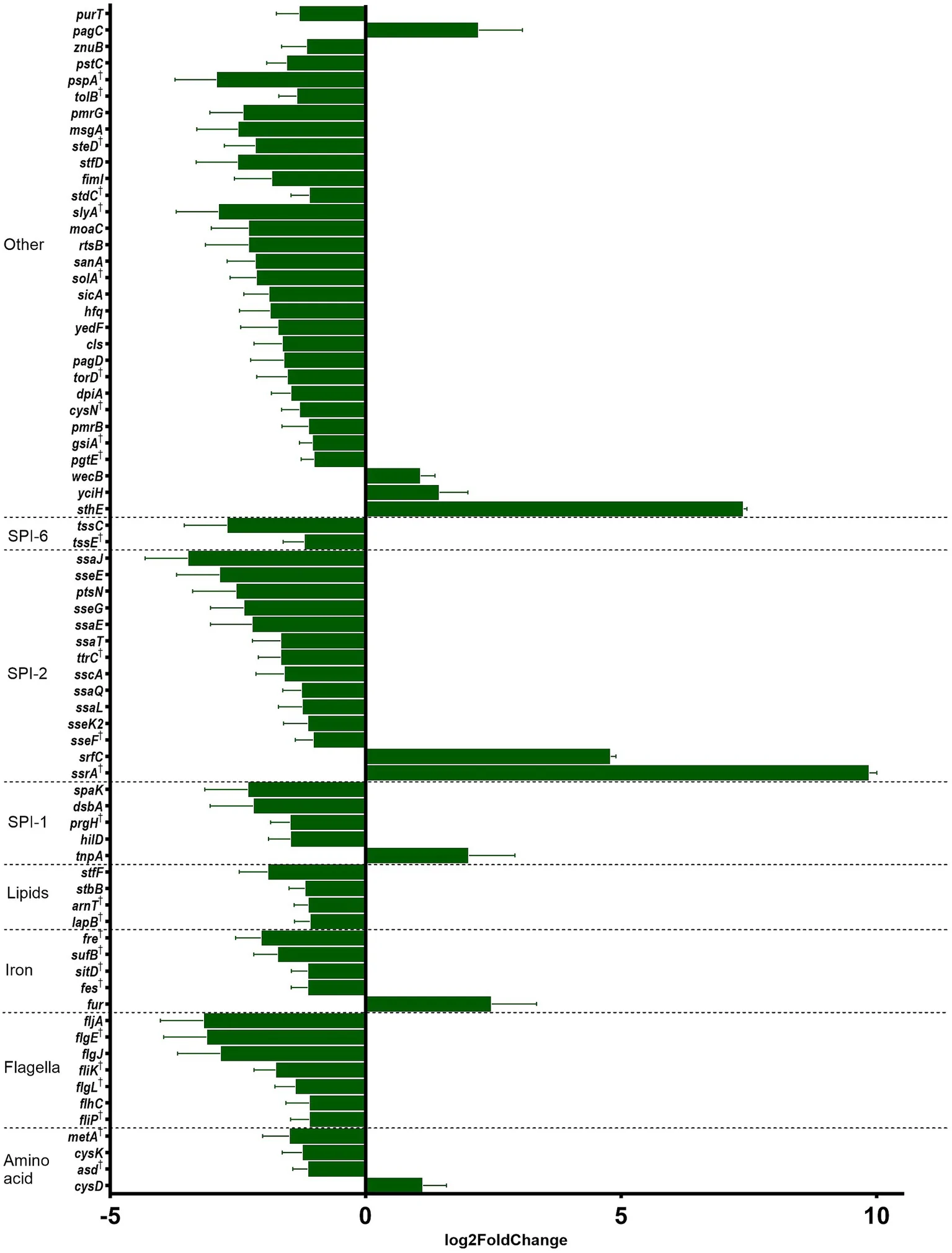
All virulence-associated genes among monophasic *Salmonella* Typhimurium (green) isolates compared to *Salmonella Heidelberg* isolates from the selected DEG list.

**Figure 8:**
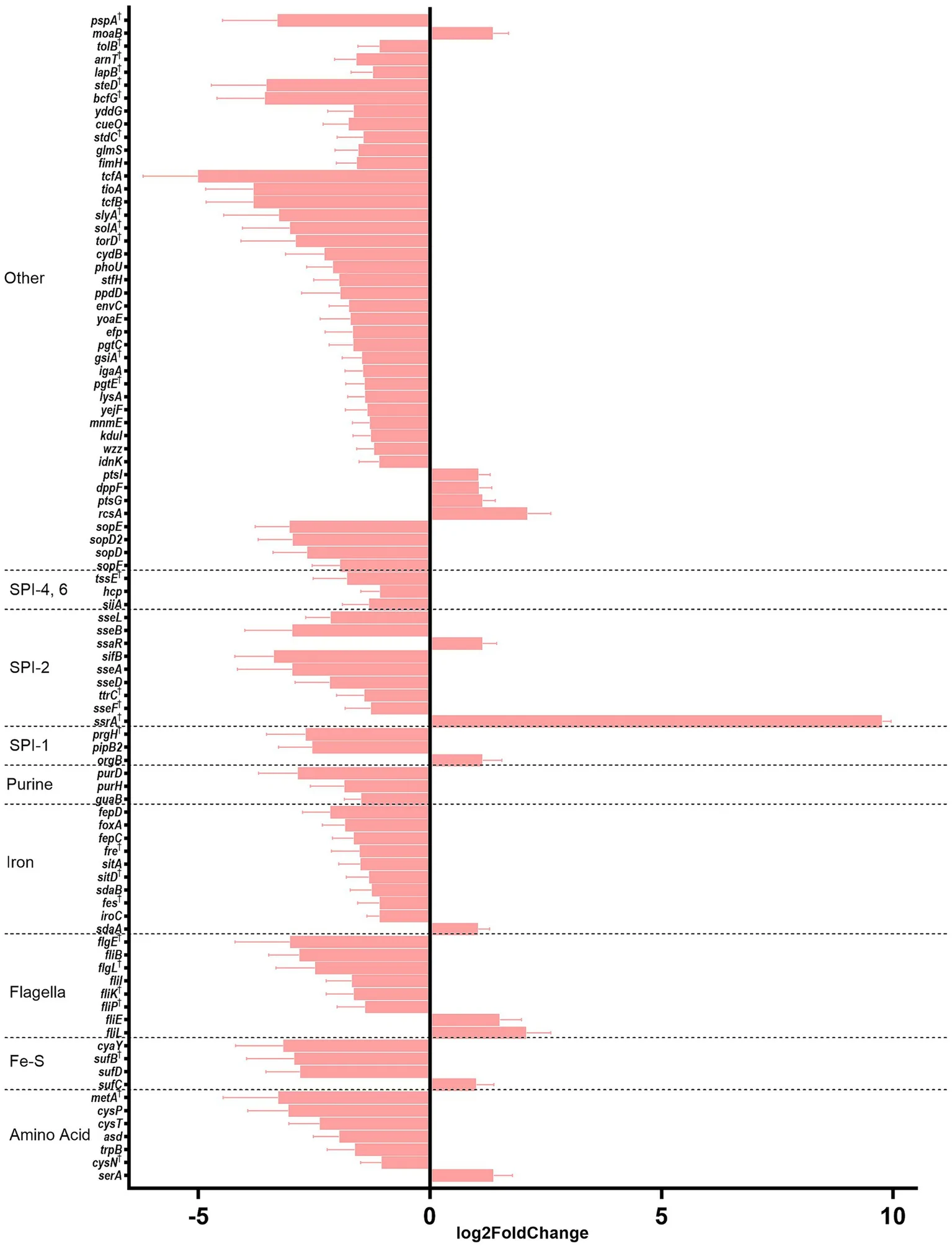
All virulence-associated genes among *Salmonella* Typhimurium (pink) isolates compared to *Salmonella Heidelberg* isolates from the selected DEG list.

### AMR, biofilm, quorum sensing, heat shock, DNA repair, heavy metal DEG

Multiple AMR genes were downregulated in *Salmonella* Typhimurium isolates compared with *Salmonella* Heidelberg isolates (Figure 5A); these included the quinolone-binding genes *parE* and *parC*, the penicillin-binding protein genes *pbp4b* and *dacD*, and the efflux pump genes *macA*, *mdsC*, *acrB*, and *marA*, although *mdtH* was upregulated in *Salmonella* Typhimurium. Biofilm formation-associated genes (Figure 5B), *seqA* and the adenylyl cyclase gene *adrA*, as well as *wzb*, were upregulated in *Salmonella* Typhimurium, while *ygiD*, *fimZ*, *wcaIM*, and *bssR* (anti-biofilm gene) were downregulated compared with *Salmonella* Heidelberg. Monophasic *Salmonella* Typhimurium exhibited downregulation of *csgA* and *csgE*, *wrbA*, *wcaE* and *wcaI*, *gmd*, and *mcbR*, but upregulation of *wzb*, *wcaB*, and *bcsF* compared with *Salmonella* Heidelberg. The quorum-sensing-associated gene *qseB* was upregulated in *Salmonella* Typhimurium, while *luxS* was downregulated compared with *Salmonella* Heidelberg; monophasic *Salmonella* Typhimurium had no DEGs associated with quorum sensing (Figure 5C). The heat-shock proteins *ppiB* and *dnaJ* were downregulated in monophasic *Salmonella* Typhimurium. *Salmonella* Typhimurium displayed variable expression of its two DE heat-shock genes, with *ibpA* being upregulated, while *hslO*, which is also involved in the stress response, was downregulated. DNA repair-related genes were also downregulated in both *Salmonella* Typhimurium and monophasic *Salmonella* Typhimurium compared with *Salmonella* Heidelberg. Despite the fact that the monophasic *Salmonella* Typhimurium isolates harbored a number of acquired heavy metal tolerance genes, *arsC* for arsenic tolerance, as well as *golS* (gold tolerance), *zraS* (zinc tolerance), and *cueR* (copper tolerance) were downregulated, while only *arsR*, encoding the arsenic tolerance regulator, was upregulated (Figure 5D). *Salmonella* Typhimurium downregulated all evaluated heavy metal tolerance genes.

### Stress response DEG

The same general pattern of gene downregulation compared with *Salmonella* Heidelberg was also observed among general stress response genes (Figure 6). Acid-tolerance-associated genes *asr* and *rstB* were downregulated in monophasic *Salmonella* Typhimurium compared with *Salmonella* Heidelberg, as were *dmsB* (anaerobic stress) and *bhsA*, *clpS*, and *uspF* (general stress) genes. However, the stationary-phase general stress response master regulator *rpoS* was upregulated in monophasic *Salmonella* Typhimurium. Oxidative stress genes *sufA*, *mntP*, *ychF*, *yfcG*, and *soxR* were also downregulated, as was the osmoprotection gene *opgG*. Finally, *fadK*, *pepT*, and *phoB* (starvation stress), and *cgtA* (UV survival) were also downregulated, as were *pth*, encoding a negative regulator of persister formation, and *ompX*, *ompD*, *yhcN*, and *ridH* were likewise downregulated. Upregulated genes in monophasic *Salmonella* Typhimurium, besides *rpoS*, included *ripR*, which contributes to survival in macrophages, *napA* (starvation stress), and *cpxR* (envelope stress and biofilm regulation).

Fewer stress tolerance genes were differentially expressed between *Salmonella* Typhimurium and *Salmonella* Heidelberg. However, expression patterns were broadly similar to those for monophasic *Salmonella* Typhimurium. The acid-tolerance gene *cadB* was upregulated, but *rstB* was downregulated, similar to the pattern in monophasic *S.* Typhimurium. The cold-shock protein gene c*spE* was also downregulated, while *elaB* was upregulated. The CRISPR–Cas system gene *cas5e* and the phage shock gene *pspF* were downregulated, as was the osmoprotection gene *proW*. Genes *pth*, *yhcN*, and *queE*, as well as the oxidative stress genes *cysJ*, *ychF*, *yfcG*, *greA*, *katE*, and *yqhD* were all downregulated. Overall, *Salmonella* Heidelberg displayed higher expression of most evaluated genes, despite the implementation of standard library-size normalization functions in DESeq2.

### Virulence DEG

Virulence-related genes were also upregulated in the *Salmonella* Heidelberg isolates compared with both monophasic *Salmonella* Typhimurium and *Salmonella* Typhimurium (Figures 7, 8). Genes associated with SPI-6 (*tssCE*) were upregulated in Heidelberg, along with SPI-2 genes (*ssaEJLQT*, *sseEFGK2*, *sscA*, *ptsN*, and *ttrC*), and SPI-1 genes (*spaK*, *dsbA*, *prgH*, and *hilD*). Notably, the SPI-1 gene *tnpA* and the SPI-2 genes *srfC* and *ssrA* were upregulated in monophasic *Salmonella* Typhimurium, with the latter expressed at a nearly 10-fold higher level than in *Salmonella* Heidelberg. Lipid-associated genes (*stfF*, *stbB*, *arnT*, *lapB*) and iron-associated genes (*fre*, *sufB*, *sitD*, *fes*) were downregulated among monophasic *Salmonella* Typhimurium isolates, though *fur*, a master regulator of iron uptake and metabolism, was upregulated (Mey et al., 2021). As anticipated, flagellar-associated genes including *fljA*, *flgEJL*, *fliKP*, and *flhC* were downregulated in monophasic *Salmonella* Typhimurium isolates, concordant with the lack of phase variation. Amino acid biosynthesis-associated genes *metA*, *cysKD*, and *asd* were all downregulated among monophasic *Salmonella* Typhimurium isolates. Other virulence-associated genes upregulated among monophasic *Salmonella* Typhimurium isolates included *pagC* (promotes survival in macrophages under acidic conditions), *wecB* (critical for infection in the poultry-associated serovar *Salmonella* Gallinarum), *yciH* (translation initiation factor), and *sthE* (fimbrial protein, fimbrial assembly). However, the majority of virulence-associated genes were upregulated in *Salmonella* Heidelberg isolates, most notably including *msgA* (survival in macrophages), *slyA* (master regulator), and *steD* (immune evasion).

A greater number of virulence-associated genes were differentially expressed between *Salmonella* Typhimurium and *Salmonella* Heidelberg, though most were upregulated in the *Salmonella* Heidelberg isolates. Genes *serA* (amino acid biosynthesis), *suf* (Fe-S cluster assembly), *fljL* and *fliE* (flagella-associated), *sdaA* (iron), *orgB* (SPI-1), and *ssrR* and *ssrA* (SPI-2) were upregulated in *Salmonella* Typhimurium isolates, with *ssrA*, encoding a regulator mediating intracellular survival, expressed at a nearly 10-fold higher level than in *Salmonella* Heidelberg. However, numerous SPI-2 genes were upregulated in *Salmonella* Heidelberg, including *sseABDFL*, *sifB*, and *ttrC*, as well as the SPI-1 gene *prgH* and the SPI-2-translocated effector gene *pipB2*. Genes *tssE* and *hcp* (SPI-6, encoding components of the type VI secretion system), and *siiA* (SPI-4) were also upregulated. Genes associated with iron acquisition, including *sitAD*, *fepCD*, *iroC*, *fes*, and *fre*, were all upregulated, as well as metabolic genes involved in amino acid biosynthesis (m*etA*, *cysNPT*), purine biosynthesis, and iron–sulfur clustering (s*ufBC*).

## Discussion

### The global upregulation of genes encoding SPI-1-associated functions, along with increased expression of motility and iron-acquisition genes in *Salmonella* Heidelberg, suggests enhanced invasion capabilities

Transcript abundances of SPI-1 genes were higher in *Salmonella* Heidelberg isolates than in both *Salmonella* Typhimurium and the monophasic variant. Both SPI-1 and SPI-2 encode the T3SS, but SPI-1 effectors are associated with host cell invasion and infection, while SPI-2 effectors are primarily associated with survival (Jennings et al., 2017). The SPI-1-associated transcriptional regulator *hilD* was upregulated in *Salmonella* Heidelberg compared with monophasic *Salmonella* Typhimurium, while *Salmonella* outer protein genes (sopD, sopD2, sopE, and sopF) were upregulated compared with Typhimurium. Both sets of genes contribute to invasion (Lou et al., 2019) and pathogenicity (Shah, 2014). The gene *prgH*, encoding the outer ring of the T3SS needle complex (Lou et al., 2019), was upregulated in *Salmonella* Heidelberg compared with both monophasic *Salmonella* Typhimurium and *Salmonella* Typhimurium, while *Salmonella* invasion chaperone A (*sicA*) was upregulated only compared with monophasic *Salmonella* Typhimurium. Shah previously found these SPI-1 genes to be upregulated in highly pathogenic *Salmonella* Enteritidis strains (Shah, 2014), and *sicA* was expressed at lower levels in *Salmonella* Cerro isolates when compared with *Salmonella* Javiana and *Salmonella* Typhimurium isolates (Cohn et al., 2021). However, it is important to note that these two studies used strains in the exponential growth phase, and differences in growth conditions and methodologies necessitate caution when comparing findings. SPI-1 genes in the latter study were broadly expressed at higher abundances in *Salmonella* Javiana and *Salmonella* Typhimurium, and this was attributed to *Salmonella* Cerro’s relatively lower pathogenicity (Cohn et al., 2021) Outside the SPIs, the RNA chaperone gene *hfq* was upregulated in *Salmonella* Heidelberg compared with monophasic *Salmonella* Typhimurium; its role is to post-transcriptionally regulate approximately 20% of *Salmonella* genes (Behera et al., 2016). In addition, *hfq* is associated with enhanced virulence and stress tolerance, with a deletion resulting in attenuated stress tolerance (Behera et al., 2016).

Motility-associated genes were also upregulated in *Salmonella* Heidelberg, including the flagellar genes *fliK*, which controls the length of the flagellar hook, membrane protein gene *fliP*, hook gene *flgE*, flagellar hook-associated protein gene *flgL*, and the rod assembly protein gene *flgJ* (compared with monophasic *Salmonella* Typhimurium only). The master regulator gene *flhC* was upregulated in the Heidelberg isolates compared with monophasic *Salmonella* Typhimurium, while *fliI* and *fliB* were upregulated when compared only with *Salmonella* Typhimurium. As the flagellar-specific T3SS export apparatus components are evolutionarily and functionally linked to virulence (Erhardt et al., 2014), it is critical to value flagellar contributions to overall motility and pathogenic capacity. In Shah’s evaluation of low- and high-pathogenicity *Salmonella* Enteritidis strains, the latter upregulated *fliK*, *flgE*, and *fljL* (Shah, 2014), which were downregulated in both *Salmonella* Typhimurium variants in this study. The genes *fliB* and *fliI* were also downregulated in both the Shah low-pathogenicity strain (Shah, 2014) and our *Salmonella* Typhimurium isolates; however, *fliE* (essential adaptor for flagellum assembly and function) (Hendriksen et al., 2021) and *fliL* were upregulated in our *Salmonella* Typhimurium isolate. The heterogeneous expression of flagellar genes between *Salmonella* Heidelberg and *Salmonella* Typhimurium was surprising, though the general upregulation of flagellar genes among *Salmonella* Heidelberg isolates supports this serovar’s known higher virulence (Jones et al., 2008).

The increased transcriptome abundance of iron-acquisition and metabolism-associated genes in *Salmonella* Heidelberg further suggests enhanced fitness contributing to pathogenicity. The master regulator *fur*, which acts as a repressor in the presence of ferrous iron, was upregulated in monophasic *Salmonella* Typhimurium when compared with *Salmonella* Heidelberg (Dominguez-Acuna and Garcia-Del Portillo, 2022). Higher expression of Fur, resulting in the repression of iron acquisition processes, may indicate stress conditions, such as oxidative stress, in which iron is acquired from alternative sources (Frawley and Fang, 2014). Other iron and iron–sulfur (Fe–S) cluster-associated genes were all upregulated in *Salmonella* Heidelberg compared to monophasic *Salmonella* Typhimurium, including *pmrG* (senses high iron levels, remodels LPS for survival); *fes* (enterobactin esterase for release of iron within the cytoplasm); *sufB* (component of the *suf* operon for Fe–S clusters); and *sitD* (component of the *sit* operon for iron/manganese transport). The genes *cas5e* (CRISPR *cas* gene, which has been associated with increased iron metabolism and heat tolerance; Sarjit et al., 2021), *cyaY* (iron chaperone supporting Fe–S cluster biosynthesis), *fepCD*, *foxA*, *sdaB*, and *iroC* (membrane transporter) were upregulated in *Salmonella* Heidelberg compared with *Salmonella* Typhimurium. Iron uptake is critical for persistence in the vacuoles of host cells (Dominguez-Acuna and Garcia-Del Portillo, 2022), and Fe–S clusters are key cofactors for metabolic, regulatory, and stress response proteins (Shah, 2014). The *suf* operon specifically has been found to be downregulated in low-pathogenicity *Salmonella* Enteritidis strains (Shah, 2014), emphasizing its role in enhanced virulence.

The higher transcript abundance of metabolism-associated genes, alongside pathway overrepresentation data, indicates that *Salmonella* Heidelberg’s enhanced metabolic processes may contribute to its pathogenic strategies. PANTHER overrepresentation analysis determined that metabolic process pathways were significantly enriched, including the upregulation of the genes *cydB* (aerobic respiration), *torD* (anaerobic respiration), and *ttrE* (tetrathionate respiration), indicating a potential increase in metabolic fitness. Previous work by Shah et al. demonstrated that a low-pathogenicity *Salmonella* Enteritidis strain exhibited reduced nutrient metabolism (Shah, 2014). Overall, this work suggests that the *Salmonella* Heidelberg isolates in this study demonstrate enhanced virulence profiles supported by robust metabolic activity and invasion machinery.

### Stationary-phase cultures of monophasic *Salmonella* Typhimurium and *Salmonella* Typhimurium expressed select genes associated with the stress response and SPI-2, indicating enhanced survival and an alternative pathogenic strategy

Genes involved in responses to various environmental stressors, such as acid and osmotic stress, along with select SPI-2 genes associated with survival in the *Salmonella*-containing vacuole, suggest a pathogenic strategy emphasizing stress adaptation and intracellular survival rather than enhanced invasion. Stationary-phase conditions are known to influence virulence through upregulation of SPI-2 pathways and the downregulation of SPI-1 pathways through complex regulatory networks involving *ssrAB* and *hilD*, respectively (Bustamante et al., 2008). Isolates in this study were grown under identical conditions to stationary phase, and expression patterns may have been similarly impacted across all strains; however, differential expression among serovars reflects strain- and serovar-specific transcriptional responses. Despite the global upregulation of SPI-2 genes in *Salmonella* Heidelberg isolates, understanding the gene expression patterns of monophasic *Salmonella* Typhimurium and *Salmonella* Typhimurium strains relating to fitness and pathogenic strategies is critical, as these outbreak-associated strains also caused large and widespread outbreaks. Most notably, *ssrA* (SPI-2) and *cpxR* (stress response, biofilm) were upregulated in both monophasic *Salmonella* Typhimurium and *Salmonella* Typhimurium strains when compared with *Salmonella* Heidelberg, while *rpoS* was upregulated in monophasic *Salmonella* Typhimurium compared with *Salmonella* Heidelberg. The gene *ssrA* encodes a tmRNA, acting as both a tRMA and an mRNA to tag partially synthesized proteins for degradation, and is required for virulence (Julio et al., 2000). Previously, *ssrA* was found to be upregulated in a low-gravity-induced stress state, though this was alongside SPI-1 invasion-specific genes (Barrila et al., 2022). Numerous SPI-2 genes associated with both survival and immune function, including *sseFG*, *pipB_2_*, *sseK_2_*, and *steD* (core effectors highly conserved across intestinal serovars); *sopD_2_* (inhibitor); and *sifB* (effector less conserved), were upregulated in *Salmonella* Heidelberg when compared with monophasic *Salmonella* Typhimurium, *Salmonella* Typhimurium, or both. This indicates that *Salmonella* Heidelberg may possess a superior ability to survive in both epithelial cells and macrophages, which aligns with its increased invasive illness rate (Jennings et al., 2017; Jones et al., 2008). Overall, both SPI-1 and SPI-2 genes were upregulated in *Salmonella* Heidelberg, supporting this serovar’s higher virulence and invasiveness, while both Typhimurium variants demonstrated a transcriptomic profile oriented toward stress response and long-term survival.

The increased transcript abundance of *rpoS* in monophasic *Salmonella* Typhimurium isolates compared with *Salmonella* Heidelberg isolates indicates enhanced stress tolerance. RpoS modulates numerous physiological mechanisms and stress tolerances, including tolerance to acid stress (Guillén et al., 2021). The acid-stress-associated gene *pagC* was also upregulated in monophasic *Salmonella* Typhimurium compared with *Salmonella* Heidelberg. In contrast, only the acid stress genes *cadB* and *asrB* were upregulated in *Salmonella* Typhimurium compared with *Salmonella* Heidelberg, with no significant difference in *rpoS* expression. In addition, RpoS effectors including *hfq* and *yedADF* were downregulated when compared to *Salmonella* Heidelberg (Dong and Schellhorn, 2010). However, because RpoS has broad cellular impacts, elucidating the complexities of its regulatory networks and effectors remains challenging. Previous research has yielded mixed results regarding whether *rpoS* expression enhances or hinders invasion and lethality (Guillén et al., 2021). Furthermore, the genes *napA* (expressed in response to low nitrate concentrations) and *ripR* (itaconate response regulator, intracellular survival) were upregulated among monophasic *Salmonella* Typhimurium isolates when compared with *Salmonella* Heidelberg. As the development of stress resistance can impose a fitness cost, enhanced stress tolerance among the *Salmonella* Typhimurium variants may explain their relative decrease in gene expression associated with invasion and metabolism when compared with *Salmonella* Heidelberg (Guillén et al., 2021). Previous research by Park et al. found environmental adaptation strategies for modulating virulence in produce contaminated with *Salmonella* (Park et al., 2020). This study determined that upon contact with plant tissue, both epiphytic and planktonic bacterial cells showed upregulation of SPI-1 and downregulation of SPI-2 genes (Park et al., 2020), which they interpreted as a cellular reprogramming response altering virulence, in addition to metabolic shifts (Park et al., 2020).

Overall, the observed expression patterns suggest that enhanced stress-adaptation pathways may have supported the fitness of monophasic *Salmonella* Typhimurium and *Salmonella* Typhimurium strains, allowing them to withstand environmental stressors such as the acid and osmotic stresses encountered during food processing. This increased stress tolerance may have contributed to their ability to persist across the food chain and cause foodborne outbreaks. In contrast, *Salmonella* Heidelberg exhibited greater expression of invasion- and virulence-associated pathways, reflecting a distinct pathogenic strategy.

### Heat stress and biofilm formation genes showed variable expression profiles by serovar, concordant with phenotypic assays

Previous phenotypic assay results demonstrated that, overall, *Salmonella* Heidelberg isolates had the longest survival under 55 °C heat shock conditions, ranging from 45 to 60 min depending on the isolate (FML-M2-0001: 45 min; FML-M2-0030: 45 min; and FML-M2-0031: 60 min). However, the average *D-*value of initial heat inactivation (1.64 min, calculated from time points 0–9 min rather than time points 0–60 min rather than time points 0–60 min) among the *Salmonella* Heidelberg isolates was lower than both other serovars (monophasic *Salmonella* Typhimurium: 3.36 min, *Salmonella* Typhimurium: 1.97 min) (Martin et al., 2026). *Salmonella* Heidelberg upregulated genes *ppiB* and *dnaJ* when compared to monophasic *Salmonella* Typhimurium, and *hslO* and *fkpA* when compared with *Salmonella* Typhimurium. Increased transcript abundance of these heat tolerance-associated genes (Dawoud et al., 2017; Etter et al., 2019; Kumawat et al., 2020; Qiu et al., 2023), along with phenotypic assays, indicates enhanced heat tolerance, contributing to the strain’s overall virulence. However, research has shown that repeated heat shock alongside loss-of-function mutations in *dnaJ* increased heat tolerance but was accompanied by attenuated growth and virulence, indicating that the relationship between stress tolerance and virulence is complex (Berdejo et al., 2024). Dawoud et al. (2017) reported that a host body temperature higher than the 37 °C human host temperature (i.e., poultry, 42 °C; swine, 39 °C) could activate thermal stress mechanisms, allowing for easier colonization of host tissues, which may contribute to observed differences in intrinsic thermal adaptation based on host species.

The heat tolerance of *Salmonella* Heidelberg isolates, alongside the upregulation of SPI-1 genes and certain metabolic pathways, has been previously observed. Sarjit et al. found that isolates with upregulated *cas5e* exhibited both increased iron metabolism and increased heat tolerance (Sarjit et al., 2021), while Qiu et al. (2023) determined that metabolic pathways were upregulated in heat stress isolates. In addition, poultry outbreak-associated *Salmonella* Heidelberg revealed that heat-tolerant isolates upregulated SPI-1 virulence-associated genes, though *sicA*, *sopBE2*, and *prgH* were downregulated during heat stress compared with the stationary phase (Etter et al., 2019). This combination of enhanced fitness attributes may have contributed to the high infection and invasive illness rates of these outbreaks.

Conversely, *ibpA* upregulation in the *Salmonella* Typhimurium isolate derived from the peanut butter outbreak may have contributed to desiccation tolerance (Oliveira et al., 2026). Desiccation tolerance in low-moisture foods, including peanut butter and related products, is commonly reported (Pereira et al., 2020), and *ibpA* has been associated with both desiccation stress and heat stress (Oliveira et al., 2026; Qiu et al., 2023). Though phenotypic assays were not conducted to evaluate desiccation tolerance among isolates, the survival of *Salmonella* Typhimurium for up to 45 min of 55 °C heat shock conditions, alongside the upregulation of select heat- and desiccation-tolerance genes, may have contributed to the survival of *Salmonella* and the overall lethality of the 2008–2009 peanut butter outbreak.

Biofilm-associated gene expression was generally upregulated in *Salmonella* Heidelberg isolates, concordant with previously conducted phenotypic assays demonstrating that the Heidelberg isolates had the strongest biofilm-forming ability averaged across all conditions (refrigeration temperature and room temperature, nutrient abundance and nutrient depletion) (Martin et al., 2026). Notable genes upregulated in *Salmonella* Heidelberg isolates that contribute to biofilm formation included *wcaIM* and *fimZ* when compared with *Salmonella* Typhimurium, and *csgAE*, *wcaEI*, *mcbR*, and *gmd* when compared with monophasic *Salmonella* Typhimurium. The upregulation of curli genes (*csg* cluster) contributes to adhesion and biofilm formation, while O-antigen capsule genes (*gmd*) and numerous genes associated with colanic acid production (*wca* cluster) are attributed to the biofilm-mediated response to oxidative stress (Aleksandrowicz et al., 2023). Overall, the upregulation of biofilm-associated genes in *Salmonella* Heidelberg isolates demonstrates a more coordinated, absolute ability to support mature biofilm structure.

In contrast, the cyclic-di-GMP-dependent regulator gene *adrA* and the colanic acid biosynthesis gene *wzb* were upregulated in the *Salmonella* Typhimurium isolates, while *wcaB*, *wzb*, and the cellulose biosynthesis gene *bcsF* were upregulated in the monophasic *Salmonella* Typhimurium isolates compared with the Heidelberg isolates. The envelope stress response regulator gene *cpxR* was upregulated in both *Salmonella* Typhimurium and monophasic *Salmonella* Typhimurium isolates. This gene responds to envelope stress and is important for biofilm formation, but it can repress curli formation and surface adhesion upon sensing stress (Jubelin et al., 2005; Shetty et al., 2019). The comparatively reduced number of colanic acid gene transcripts may suggest reduced biofilm capacity, while the increased transcript abundance of *cpxR* may indicate increased response to envelope stress that may impact curli production and overall biofilm formation. Phenotypic assays indicated reduced and less consistent biofilm formation in the Typhimurium variants, which may indicate less coordinated biofilm development than in *Salmonella* Heidelberg isolates. The upregulation of two major stress regulators, *cpxR* in both *Salmonella* Typhimurium variants and *rpoS* in *Salmonella* Typhimurium, supports the increased cellular emphasis on overall stress-response adaptations.

Furthermore, the upregulation of the autoinducer-2 synthase gene *luxS* in *Salmonella* Heidelberg isolates when compared with *Salmonella* Typhimurium may aid in biofilm formation and enhance antimicrobial resistance (Ju et al., 2018). This could support the extensive AMR profile of the *Salmonella* Heidelberg isolates. Genotypic and phenotypic resistance to aminoglycosides, bleomycin, fosfomycin, sulfonamides, and tetracycline (Etter et al., 2019; Gieraltowski et al., 2016; Hoffmann et al., 2013) was reflected in the enhanced expression of numerous genes encoding efflux pumps such as *ydeE*, *robA*, *mdsC*, *acrB*, and *macA*. The ability to form mature biofilms for bacterial survival and the development of antibiotic resistance likely further contributed to the scope of the 2011 kosher broiled chicken liver outbreak and 2013–2014 chicken outbreak, both caused by *Salmonella* Heidelberg. In addition, monophasic *Salmonella* Typhimurium isolates exhibited upregulation of the arsenic operon regulator *arsR*, along with predicted resistance to the metals copper, silver, gold, mercury, and arsenic, as well as the antibiotic classes aminoglycosides, β -lactams, sulfonamides, and tetracyclines. Previous work has demonstrated that metal tolerance gene acquisition among monophasic *Salmonella* Typhimurium isolated from pork production environments may facilitate bacterial survival and possibly co-select for antibiotic resistance genes (Bearson et al., 2020).

The observed transcriptional differences among outbreak-associated serovars may provide insight into how distinct adaptations contribute to survival across diverse food environments. Heat tolerance and biofilm-associated gene expression, and virulence pathways among *Salmonella* Heidelberg may have contributed to its fitness in poultry-associated outbreaks, while stress-associated pathways in monophasic *Salmonella* Typhimurium and *Salmonella* Typhimurium isolates may reflect adaptation to environmental challenges in their respective food processing and transmission matrices. Specifically, desiccation-associated responses in the *Salmonella* Typhimurium isolate may be relevant to survival within the low-moisture food environment associated with peanut butter, while metal-tolerance profiles in monophasic *Salmonella* Typhimurium may reflect selective pressures within swine production environments.

However, certain limitations of this study should be considered. The inclusion of avirulent or attenuated strains would have provided an additional baseline for comparison, as evaluating only highly pathogenic, outbreak-associated strains may have limited our ability to detect the full extent of DEGs and functional pathway enrichment. Furthermore, functional phenotypic validations such as Caco-2 cell invasion assays or RNA-Seq of strains in stressed conditions would have further contextualized these findings but were outside the scope of this study. Nevertheless, profiling *S. enterica* isolates in the stationary phase provided a physiologically relevant model reflecting bacterial states in food matrices, thereby allowing for meaningful comparisons among strains.

## Conclusion

This study presents a novel comparative transcriptomic evaluation of multiple outbreak-associated *S. enterica* serovars to characterize differential gene expression attributable to outbreak dynamics and phenotypic traits. Findings indicate that the outbreak-associated *Salmonella* Heidelberg strains in this study exhibited transcriptional profiles with upregulated genes involved in invasion and iron metabolism, whereas *Salmonella* Typhimurium and monophasic *Salmonella* Typhimurium showed expression profiles more strongly aligned with stress response and survival mechanisms. These transcriptomic profiles were concordant with phenotypic assays, in which *Salmonella* Heidelberg demonstrated greater biofilm-forming capabilities and prolonged survival at lethal temperatures. Overall, these findings suggest that outbreak severity and scope may have been influenced by the intrinsic phenotypic and transcriptional characteristics of the respective outbreak-associated strains. Further research is warranted to elucidate transcriptomic variation across additional serovars, ultimately facilitating serovar-specific risk assessments and targeted food safety interventions.

## Data Availability

The datasets presented in this study can be found in online repositories. The names of the repository/repositories and accession number(s) can be found in the article/Supplementary material.

## Footnotes

1
https://github.com/FelixKrueger/TrimGalore

2
http://pantherdb.org/

## Supplementary material

SUPPLEMENTARY FIGURE S1 Pathway analysis using PANTHER and gene ontology (GO) reveals GO process terms associated with DEG comparisons between monophasic *Salmonella* Typhimurium and Salmonella Heidelberg **(A)**, *Salmonella* Typhimurium and *Salmonella* Heidelberg **(B)**, and *Salmonella* Typhimurium and monophasic *Salmonella* Typhimurium **(C)**. Pathways were highly similar between comparisons 1 and 2, indicating high similarity between monophasic *Salmonella* Typhimurium and *Salmonella* Typhimurium when compared to Salmonella Heidelberg.
