# Supplementary material for: Comparative transcriptomics of outbreak-associated Salmonella Heidelberg, Typhimurium, and monophasic Typhimurium reveals differential expression of stress tolerance, virulence, and biofilm-associated genes
Source: Front Microbiol. 2026 Oct 12;17:1895105. doi: 10.3389/fmicb.2026.1895105 (PMC13696503; doi:10.3389/fmicb.2026.1895105)
Supplement: Supplementary file 1 [file Supplementary_file_1.docx]

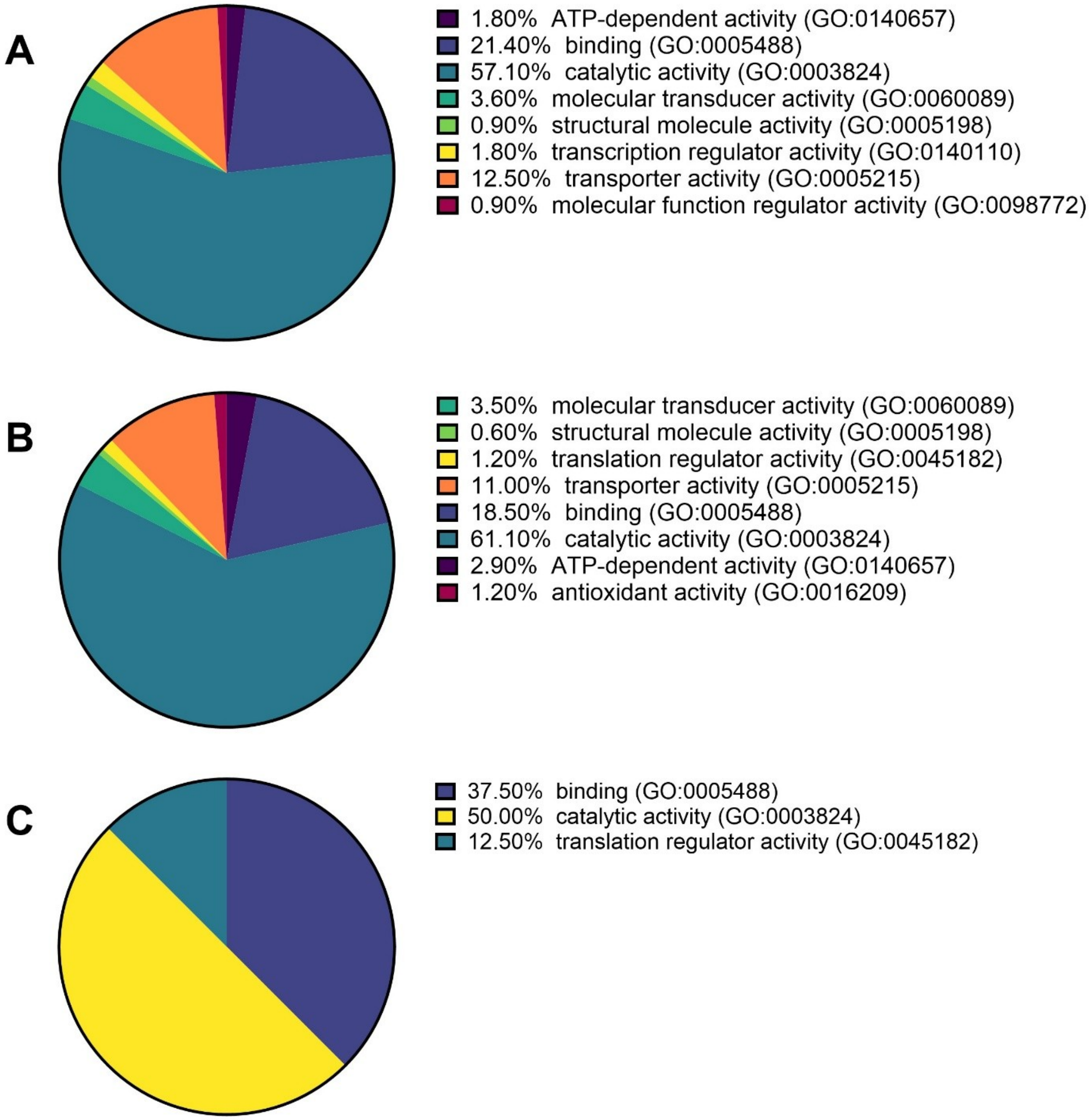
A
1.80% ATP-dependent activity (GO:0140657)
21.40% binding (GO:0005488)
57.10% catalytic activity (GO:0003824)
3.60% molecular transducer activity (GO:0060089)
0.90% structural molecule activity (GO:0005198)
1.80% transcription regulator activity (GO:0140110)
12.50% transporter activity (GO:0005215)
0.90% molecular function regulator activity (GO:0098772)
B
3.50% molecular transducer activity (GO:0060089)
0.60% structural molecule activity (GO:0005198)
1.20% translation regulator activity (GO:0045182)
11.00% transporter activity (GO:0005215)
18.50% binding (GO:0005488)
61.10% catalytic activity (GO:0003824)
2.90% ATP-dependent activity (GO:0140657)
1.20% antioxidant activity (GO:0016209)
C
37.50% binding (GO:0005488)
50.00% catalytic activity (GO:0003824)
12.50% translation regulator activity (GO:0045182)
