# Supplementary material for: Comparative transcriptomics of outbreak-associated Salmonella Heidelberg, Typhimurium, and monophasic Typhimurium reveals differential expression of stress tolerance, virulence, and biofilm-associated genes
Source: Front Microbiol. 2026 Oct 12;17:1895105. doi: 10.3389/fmicb.2026.1895105 (PMC13696503; doi:10.3389/fmicb.2026.1895105)
Supplement: Supplementary file 2 [file Table_1.docx]

**Table S1A-C. Differentially expressed genes (DEG) by pairwise comparison.** Table includes category, function, whether they were up or ↓, log2FoldChange (L2FC), and *p-adj* value. Genes are sorted by ascending L2FC (log2FoldChange).

**Table S1A. DEG among pairwise comparison 1: monophasic Typhimurium versus Heidelberg, where Heidelberg was the reference category.**

| Gene | Category^a^ | Subcategory^b^  (if applicable) | Description^c^ | Up or Down-regulated | L2FC | *p_adj_* |
| --- | --- | --- | --- | --- | --- | --- |
| *ssaJ^†^* | Virulence | SPI-2 | type III secretion apparatus lipoprotein, YscJ/HrcJ family | ↓ | -3.481027276 | 0.0001067 |
| *fljA^†^* | Virulence | Flagella | repressor of phase 1 flagellin gene | ↓ | -3.179254885 | 0.0004342 |
| *flgE* | Virulence | Flagella | flagellar hook protein FlgE | ↓ | -3.116112016 | 0.0005520 |
| *soxR* | Stress Response |  | redox-sensitive transcriptional activator SoxR | ↓ | -3.005886271 | 0.0005630 |
| *dmsB* | Stress Response | Oxidative stress response | dimethylsulfoxide reductase, chain B | ↓ | -2.95906381 | 0.0010330 |
| *pspA* | Virulence | Phage shock protein | phage shock protein A (phage shock protein A) | ↓ | -2.919809404 | 0.0008328 |
| *slyA^†^* | Virulence |  | transcriptional regulator SlyA | ↓ | -2.887948113 | 0.0010814 |
| *sseE^†^* | Virulence | SPI-2 |  | ↓ | -2.859256824 | 0.0016579 |
| *mutM* | DNA Repair |  |  | ↓ | -2.851519438 | 0.0002634 |
| *flgJ* | Virulence | Flagella | flagellar rod assembly protein/muramidase FlgJ | ↓ | -2.842648716 | 0.0017118 |
| *tatA* | AMR |  | Sec-independent protein translocase protein TatA | ↓ | -2.781069452 | 0.0022845 |
| *tssC^†^* | Virulence | SPI-6 | type VI secretion system contractile sheath large subunit | ↓ | -2.714024074 | 0.0027853 |
| *mdtI* | AMR |  | multidrug transport protein (spermidine export protein MdtI) | ↓ | -2.703682145 | 0.0020192 |
| *csgA^†^* | Biofilm | Curli formation | curli subunit of csgBAC operon | ↓ | -2.697375249 | 0.0029451 |
| *mntP* | Stress Response | Nitric oxide stress response |  | ↓ | -2.65286464 | 0.0034289 |
| *yhcN* | Stress Response |  | membrane protein | ↓ | -2.552631667 | 0.0011913 |
| *ptsN* | Virulence | SPI-2 | PTS IIA-like nitrogen-regulatory protein PtsN | ↓ | -2.538016009 | 0.0059938 |
| *stfD^†^* | Virulence | Fimbrae | periplasmic fimbrial chaperone StfD (gi\|3747031) | ↓ | -2.508187613 | 0.0043046 |
| *msgA^†^* | Virulence | Invasion | macrophage survivial gene | ↓ | -2.498315467 | 0.0044072 |
| *wrbA* | Biofilm |  | NAD(P)H:quinone oxidoreductase, type IV | ↓ | -2.451203148 | 0.0052324 |
| *pmrG* | Virulence | LPS, iron |  | ↓ | -2.398592631 | 0.0006233 |
| *sseG^†^* | Virulence | SPI-2 | secretion system effector SseG | ↓ | -2.38972634 | 0.0006453 |
| *ydeE* | AMR | Efflux pump | efflux MFS transporter | ↓ | -2.350447472 | 0.0028719 |
| *bhsA* | Stress Response | Oxidative stress response, biofilm | also YhcN, YcfR in e. coli | ↓ | -2.333518959 | 0.0031349 |
| *spaK^†^* | Virulence | SPI-1 | SPI-1 type III secretion system chaperone SpaK | ↓ | -2.306534514 | 0.0118988 |
| *moaC* | Virulence |  | molybdenum cofactor biosynthesis protein C | ↓ | -2.297629384 | 0.0034385 |
| *rtsB* | Virulence | Motility, invasion | GerE family regulatory protein | ↓ | -2.294055665 | 0.0124911 |
| *yedD* | AMR | Efflux pump | Uncharacterized lipoprotein YedD | ↓ | -2.267527115 | 0.0003943 |
| *wcaE^†^* | Biofilm | Colanic acid | Colanic acid biosynthesis glycosyltransferase WcaE | ↓ | -2.233429378 | 0.0045017 |
| *ssaE^†^* | Virulence | SPI-2 | type III secretion system protein, YseE family (secretion system chaperone SsaE) | ↓ | -2.223160624 | 0.0122630 |
| *yccA* | AMR, Stress Response |  | TEGT family carrier | ↓ | -2.213640907 | 0.0124186 |
| *csgE^†^* | Biofilm | Curli formation | curli production assembly/transport component CsgE | ↓ | -2.203484387 | 0.0160782 |
| *dsbA^†^* | Virulence | SPI-1 | periplasmic protein disulfide isomerase I | ↓ | -2.203118926 | 0.0168532 |
| *steD^†^* | Virulence | Immune evasion |  | ↓ | -2.165389705 | 0.0008044 |
| *sanA* | Virulence |  | Vancomycin sensitivity protein | ↓ | -2.160925447 | 0.0002713 |
| *solA^†^* | Virulence |  | N-methyltryptophan oxidase | ↓ | -2.140053126 | 0.0001023 |
| *asr* | Stress Response | Acid stress | acid shock protein | ↓ | -2.111652024 | 0.0009696 |
| *opgB* | Stress Response | Biocide tolerance |  | ↓ | -2.093942626 | 0.0030311 |
| *pth* | Stress Response | Growth | peptidyl-tRNA hydrolase (peptidyl-tRNA hydrolase, PTH1 family) | ↓ | -2.074201332 | 0.0004529 |
| *fre* | Virulence |  | NAD(P)H-flavin reductase enzyme | ↓ | -2.051825881 | 0.0001115 |
| *ppiB* | Heat shock, Stress Response | Heat shock protein | peptidylprolyl isomerase (peptidylprolyl cis-trans isomerase B (cyclophilin B)) | ↓ | -2.041907998 | 0.0095940 |
| *arsC* | Heavy Metal | Arsenic tolerance | arsenate reductase | ↓ | -1.982568955 | 0.0048595 |
| *pepT* | Stress Response | Low O2 response | aminotripeptidase | ↓ | -1.958333147 | 0.0054347 |
| *robA* | AMR | Efflux pump | MDR efflux pump AcrAB transcriptional activator RobA | ↓ | -1.947593914 | 0.0026464 |
| *mcbR* | Biofilm | Colanic acid | Colanic acid/biofilm transcriptional regulator McbR | ↓ | -1.937917567 | 0.0028750 |
| *stfF^†^* | Virulence | Lipids | Putative minor fimbrial subunit | ↓ | -1.922079304 | 0.0012291 |
| *sicA^†^* | Virulence | Type III Secretion | type III secretion low calcium response chaperone LcrH/SycD | ↓ | -1.896240993 | 0.0003191 |
| *uspF* | Stress Response |  | universal stress protein F | ↓ | -1.888913437 | 0.0336753 |
| *hfq^†^* | Virulence |  | RNA chaperone Hfq (host factor-I protein) | ↓ | -1.876232583 | 0.0035064 |
| *yfcG* | Stress response | Oxidative stress response | thiol:disulfide oxidoreductase | ↓ | -1.844624679 | 0.0042163 |
| *fimI^†^* | Virulence | Fimbrae | fimbrial protein internal segment | ↓ | -1.841335084 | 0.0207995 |
| *rstB* | Stress Response | Motility, invasion, iron | two-component system sensor histidine kinase | ↓ | -1.833004624 | 0.0001144 |
| *ruvA* | DNA Repair |  | Holliday junction DNA helicase RuvA (holliday junction DNA helicase RuvA) | ↓ | -1.832110825 | 0.0009349 |
| *cueR* | Heavy Metal | Copper tolerance | Cu(I)-responsive transcriptional regulator | ↓ | -1.810341543 | 0.0225072 |
| *clpS* | Stress Response | Stress response |  | ↓ | -1.774488209 | 0.0001759 |
| *yebG* | DNA Repair |  | DNA damage-inducible protein YebG | ↓ | -1.765700087 | 0.0061951 |
| *fliK* | Virulence | Flagella | flagellar hook-length control protein (flagellar hook-length control protein FliK) | ↓ | -1.763880881 | 0.0001063 |
| *sufA* | Stress Response | H2O2 stress response | FeS assembly scaffold (Fe-S cluster assembly protein SufA) | ↓ | -1.762243046 | 0.0270632 |
| *mdsC* | AMR | Efflux pump |  | ↓ | -1.746824902 | 0.0001544 |
| *sufB* | Virulence | Iron | FeS assembly protein (Fe-S cluster assembly protein SufB) | ↓ | -1.723840051 | 0.0006152 |
| *yedF* | Virulence |  | putative sulfur carrier protein | ↓ | -1.720175889 | 0.0315597 |
| *ompD* | Stress Response | Low pH and O2 response | outer membrane porin protein | ↓ | -1.674628284 | 0.0058791 |
| *ruvC* | DNA Repair |  | crossover junction endodeoxyribonuclease RuvC | ↓ | -1.672739803 | 0.0026472 |
| *ychF^†^* | Stress Response | Oxidative stress response | redox-regulated ATPase | ↓ | -1.667553226 | 0.0060872 |
| *ssaT^†^* | Virulence | SPI-2 | EscT/YscT/HrcT family type III secretion system export apparatus protein | ↓ | -1.66704112 | 0.0054331 |
| *ttrC^†^* | Virulence | SPI-2 | tetrathionate reductase enzyme subunit | ↓ | -1.663242685 | 0.0004249 |
| *cls^†^* | Virulence |  | cardiolipin synthase gene | ↓ | -1.637954628 | 0.0059918 |
| *golS* | Heavy Metal | Gold tolerance | small cytoplasmic metal binding protein | ↓ | -1.61765659 | 0.0126465 |
| *pagD^†^* | Virulence |  | virulence factor | ↓ | -1.603033311 | 0.0244464 |
| *sscA^†^* | Virulence | SPI-2 | CesD/SycD/LcrH family type III secretion system chaperone | ↓ | -1.59949462 | 0.0073646 |
| *pstC^†^* | Virulence | Phosphate transport | phosphate ABC transporter permease | ↓ | -1.551439473 | 0.0001864 |
| *torD^†^* | Virulence |  | molecular chaperone | ↓ | -1.535874177 | 0.0184340 |
| *ridA* | Stress Response |  |  | ↓ | -1.530376523 | 0.0323168 |
| *metA^†^* | Virulence | Amino acid biosynthesis | homoserine O-succinyltransferase | ↓ | -1.498448835 | 0.0079026 |
| *prgH^†^* | Virulence | SPI-1 | type III secretion system protein | ↓ | -1.482704329 | 0.0002557 |
| *hilD^†^* | Virulence | SPI-1 | AraC family transcriptional regulator | ↓ | -1.473546326 | 0.0014436 |
| *dpiA^†^* | Virulence |  | response regulator | ↓ | -1.470177192 | 0.0002673 |
| *yedA* | AMR |  | drug/metabolite exporter | ↓ | -1.414570778 | 0.0011844 |
| *flgL* | Virulence | Flagella | flagellar hook-associated protein 3 (flagellar hook-associated protein 3 FlgL) | ↓ | -1.379806687 | 0.0013029 |
| *pagP* | AMR |  | antimicrobial peptide resistance and lipid A acylation protein PagP | ↓ | -1.379603739 | 0.0004875 |
| *tolB* | Virulence | Membrane | Tol-Pal system beta propeller repeat protein | ↓ | -1.353654348 | 0.0002974 |
| *purT* | Virulence | Purine biosynthesis | phosphoribosylglycinamide formyltransferase 2 | ↓ | -1.306715924 | 0.0068142 |
| *cysN* | Virulence |  | sulfate adenylyltransferase subunit | ↓ | -1.299813505 | 0.0005152 |
| *ssaQ^†^* | Virulence | SPI-2 | type III secretion system protein | ↓ | -1.260623551 | 0.0012453 |
| *cysK* | Virulence | Amino acid biosynthesis | cysteine synthase A (cysteine synthase A) | ↓ | -1.248297781 | 0.0028804 |
| *ssaL^†^* | Virulence | SPI-2 | secretion system apparatus protein SsaL | ↓ | -1.242548519 | 0.0140658 |
| *wcaI* | Biofilm | Colanic acid | colanic acid biosynthesis glycosyltransferase | ↓ | -1.226209642 | 0.0010943 |
| *ompX* | Stress Response | H2O2 stress response | outer membrane protein X | ↓ | -1.219376715 | 0.0440764 |
| *tatD* | DNA Repair |  | deoxyribonuclease TatD (TatD DNase family protein) | ↓ | -1.210335 | 0.0004361 |
| *tssE* | Virulence | SPI-6 | Type VI secretion system baseplate subunit TssE | ↓ | -1.209108703 | 0.0060872 |
| *stbB^†^* | Virulence | Lipids | fimbrial chaperone protein | ↓ | -1.191849428 | 0.0003333 |
| *phoB* | Stress Response |  | phosphate regulon transcriptional regulatory protein | ↓ | -1.185882962 | 0.0049754 |
| *fadK* | Stress Response | Dessication tolerance, starvation stress, potential heat | Medium-chain fatty-acid-coenzyme A ligase | ↓ | -1.185728832 | 0.0072348 |
| *znuB^†^* | Virulence | Zinc | zinc ABC transporter permease | ↓ | -1.158755742 | 0.0296823 |
| *sitD* | Virulence | Iron | alkaline Mn2+ ABC transporter permease | ↓ | -1.139951304 | 0.0008019 |
| *sseK2^†^* | Virulence | SPI-2 | type III secretion system effector SseK2 | ↓ | -1.138659593 | 0.0268654 |
| *asd^†^* | Virulence | Amino acid biosynthesis | aspartate-semialdehyde dehydrogenase | ↓ | -1.137459586 | 0.0002466 |
| *fes* | Virulence | Iron | enterochelin esterase | ↓ | -1.137381078 | 0.0010825 |
| *acrB* | AMR | Efflux pump | acriflavine resistance protein B (multidrug efflux pump) | ↓ | -1.130964968 | 0.0003986 |
| *arnT* | Virulence | Lipids | 4-amino-4-deoxy-L-arabinose transferase | ↓ | -1.127098922 | 0.0001127 |
| *zraS* | Heavy Metal | Zinc | sensor protein ZraS | ↓ | -1.123494059 | 0.0003360 |
| *pmrB* | Virulence |  |  | ↓ | -1.122984154 | 0.0476468 |
| *cgtA* | Stress Response | Stress response | GTPase Obg | ↓ | -1.122190089 | 0.0003121 |
| *stdC^†^* | Virulence | Colonization | fimbrial chaperone protein | ↓ | -1.106839769 | 0.0040244 |
| *flhC* | Virulence | Flagella | flagellar transcriptional activator FlhC (flagellar transcriptional activator FlhC) | ↓ | -1.103569997 | 0.0285436 |
| *fliP* | Virulence | Flagella | flagellar biosynthetic protein FliP | ↓ | -1.103320136 | 0.0057434 |
| *lapB* | Virulence | Lipids | lipopolysaccharide assembly protein | ↓ | -1.09151177 | 0.0008473 |
| *gsiA* | Virulence |  | ABC-type glutathione transporter | ↓ | -1.044751843 | 0.0001070 |
| *dnaJ* | Heat shock | Heat shock protein | DnaJ domain protein | ↓ | -1.038102193 | 0.0004307 |
| *sseF^†^* | Virulence | SPI-2 | secreted effector protein (secreted effector protein SseF) | ↓ | -1.032447838 | 0.0059612 |
| *pgtE* | Virulence |  | Outer membrane protease E | ↓ | -1.013058615 | 0.0001476 |
| *gmd* | Biofilm | Colanic acid | GDP-mannose 4,6-dehydratase | ↓ | -1.002436391 | 0.0100445 |
| *wecB* | Virulence |  | UDP-N-acetylglucosamine 2-epimerase | ↑ | 1.076112573 | 0.0003855 |
| *cpxR^†^* | Stress Response |  | transcriptional regulatory protein CpxR | ↑ | 1.083443597 | 0.0003772 |
| *cysD* | Virulence | Amino acid biosynthesis | sulfate adenylyltransferase small subunit | ↑ | 1.120608873 | 0.0268807 |
| *marA* | AMR | Efflux pump | multiple antibiotic resistance protein MarA | ↑ | 1.122029622 | 0.0196965 |
| *rpoS^†^* | Stress Response |  | RNA polymerase sigma factor | ↑ | 1.262045709 | 0.0014092 |
| *wzb* | Biofilm | Colanic acid | low molecular weight protein-tyrosine-phosphatase wzb | ↑ | 1.303738945 | 0.0032865 |
| *yciH^†^* | Virulence |  | Stress response translation initiation inhibitor | ↑ | 1.444892486 | 0.0169662 |
| *wcaB* | Biofilm | Colanic acid |  | ↑ | 1.517707245 | 0.0002797 |
| *tnpA* | Virulence | SPI-1 | IS200 transposase mRNA | ↑ | 2.023145237 | 0.0408163 |
| *ripR* | Stress Response | Membrane |  | ↑ | 2.147495003 | 0.0002861 |
| *pagC^†^* | Virulence | Acid stress |  | ↑ | 2.212428876 | 0.0183226 |
| *fur^†^* | Virulence | Iron | ferric uptake regulator | ↑ | 2.467664257 | 0.0096953 |
| *bcsF* | Biofilm | Cellulose biosynthesis |  | ↑ | 3.058975016 | 0.0009800 |
| *srfC* | Virulence | SPI-2 | putative virulence effector protein | ↑ | 4.796201437 | 0.0000000 |
| *sthE^†^* | Virulence |  | FAD assembly | ↑ | 7.394325773 | 0.0000000 |
| *napA* | Stress Response |  | periplasmic nitrate reductase, large subunit | ↑ | 8.632641064 | 0.0000000 |
| *arsR* | Heavy Metal | Arsenic tolerance | arsenic | ↑ | 9.054342219 | 0.0000000 |
| *ssrA^†^* | Virulence | SPI-2 | SPI-2 | ↑ | 9.855863291 | 0.0000000 |

^†^ Indicates that this gene is considered a virulence factor according to the FDA (https://virulence.fda.gov/data/cds/?type=g).

**Table S1B. DEG among pairwise comparison 1: Typhimurium versus Heidelberg, where Heidelberg was the reference category.**

| Gene | Category | Subcategory (if applicable) | Description | Up or Down-regulated | log2FoldChange | *p_adj_* |
| --- | --- | --- | --- | --- | --- | --- |
| *tcfA^†^* | Virulence |  | Typhi colonization factor (Tcf) | ↓ | -5.011926848 | 0.000140765 |
| *zraR* | Heavy Metal | Zinc | Transcriptional regulatory protein ZraR | ↓ | -4.157168628 | 0.00033088 |
| *cas5e* | Stress Response | Iron, potential heat shock |  | ↓ | -3.834394916 | 0.004330053 |
| *tioA* | Virulence |  |  | ↓ | -3.816100146 | 0.000947893 |
| *tcfB^†^* | Virulence |  | Typhi colonization factor (Tcf) | ↓ | -3.809019605 | 0.000948369 |
| *bcfG^†^* | Virulence | Fimbrae |  | ↓ | -3.571619625 | 0.002075314 |
| *steD^†^* | Virulence | Immune evasion |  | ↓ | -3.532687651 | 0.008869786 |
| *ruvC* | DNA Repair |  |  | ↓ | -3.455588264 | 0.010241311 |
| *sifB^†^* | Virulence | SPI-2 |  | ↓ | -3.371001341 | 0.0003329 |
| *yhcN* | Stress Response |  |  | ↓ | -3.336401851 | 0.013612621 |
| *robA* | AMR | Efflux pump |  | ↓ | -3.31314683 | 0.014178573 |
| *pspA* | Virulence | Phage shock protein | phage shock protein A (phage shock protein A) | ↓ | -3.295653864 | 0.014578179 |
| *metA^†^* | Virulence | Amino acid biosynthesis |  | ↓ | -3.280895205 | 0.015093386 |
| *slyA^†^* | Virulence |  | transcriptional regulator SlyA | ↓ | -3.262251247 | 0.016470156 |
| *ychF^†^* | Stress Response | Oxidative stress response |  | ↓ | -3.261816227 | 0.016208244 |
| *mdsC* | AMR | Efflux pump | MDS efflux pump | ↓ | -3.22019032 | 0.005941015 |
| *yfcG* | Stress response | Oxidative stress response | Probable glutathione *S*-transferase | ↓ | -3.211440114 | 0.017272086 |
| *cyaY* | Virulence | Fe-S | iron donor protein CyaY (CyaY protein) | ↓ | -3.167154275 | 0.006736574 |
| *cspE^†^* | Stress Response | Cold shock | cold shock protein CspE (cold shock protein beta-ribbon, CspA family) | ↓ | -3.14908491 | 0.027602964 |
| *yebG* | DNA Repair |  |  | ↓ | -3.133050383 | 0.020233148 |
| *ydeE* | AMR | Efflux pump |  | ↓ | -3.131461115 | 0.020460779 |
| *cysP* | Virulence | Amino acid biosynthesis |  | ↓ | -3.058450571 | 0.002059751 |
| *sopE^†^* | Virulence |  | invasion-associated secreted protein | ↓ | -3.035676253 | 0.000225178 |
| *solA^†^* | Virulence |  |  | ↓ | -3.027675275 | 0.009311603 |
| *flgE* | Virulence | Flagella |  | ↓ | -3.026930407 | 0.02551532 |
| *arsR* | Heavy Metal | Arsenic tolerance | arsenic | ↓ | -2.978173163 | 0.010764932 |
| *sseB^†^* | Virulence | SPI-2, SCV, T3SS |  | ↓ | -2.975221009 | 0.010812217 |
| *sseA^†^* | Virulence | SPI-2 |  | ↓ | -2.972681656 | 0.028819771 |
| *sopD2^†^* | Virulence |  | homologous to secreted protein SopD | ↓ | -2.970876797 | 0.000315495 |
| *sufB* | Virulence | Fe-S | FeS assembly protein (Fe-S cluster assembly protein SufB) | ↓ | -2.930645202 | 0.012345207 |
| *torD^†^* | Virulence |  |  | ↓ | -2.900187027 | 0.032436125 |
| *purD^†^* | Virulence | Purine biosynthesis |  | ↓ | -2.857644367 | 0.002752772 |
| *fliB* | Virulence | Flagella |  | ↓ | -2.825933267 | 0.000101342 |
| *bcsZ* | Biofilm | Cellulose biosynthesis |  | ↓ | -2.818717338 | 0.015891742 |
| *sufD* | Virulence | Fe-S | FeS assembly protein (Fe-S cluster assembly protein SufD) | ↓ | -2.811054653 | 0.000612641 |
| *greA^†^* | Stress Response | Oxidative stress response | transcription elongation factor GreA | ↓ | -2.800924563 | 0.039665191 |
| *pth* | Stress Response |  | peptidyl-tRNA hydrolase (peptidyl-tRNA hydrolase, PTH1 family) | ↓ | -2.769540523 | 0.017704725 |
| *ruvA* | DNA Repair |  |  | ↓ | -2.720829252 | 0.019756182 |
| *hslO* | Stress Response |  |  | ↓ | -2.692949577 | 0.000209771 |
| *prgH^†^* | Virulence | SPI-1 | cell invasion protein | ↓ | -2.690754001 | 0.004598347 |
| *sopD^†^* | Virulence |  |  | ↓ | -2.655917415 | 0.001288677 |
| *fimZ^†^* | Virulence | SPI-1 |  | ↓ | -2.571612126 | 0.007013802 |
| *pipB2* | Virulence | SPI-1 | effector protein pipB2 (Secreted effector protein PipB2) | ↓ | -2.542472469 | 0.002001978 |
| *flgL* | Virulence | Flagella |  | ↓ | -2.485044379 | 0.009490232 |
| *macA* | AMR | Efflux pump | macrolide-specific efflux protein MacA | ↓ | -2.457381985 | 0.010041195 |
| *cysT* | Virulence | Amino acid biosynthesis |  | ↓ | -2.382113932 | 0.001495349 |
| *cydB* | Virulence |  | Cytochrome d ubiquinol oxidase subunit II | ↓ | -2.281193198 | 0.017034988 |
| *rcnR* | Heavy Metal | Cobalt, nickel tolerance | also YohL | ↓ | -2.270744584 | 0.017913859 |
| *sseD^†^* | Virulence | SPI-2 |  | ↓ | -2.167577418 | 0.010472678 |
| *fepD* | Virulence | Iron | iron transporter FepBDGC | ↓ | -2.153363467 | 0.001401023 |
| *sseL* | Virulence | SPI-2, T3SS, SCV |  | ↓ | -2.149131497 | 0.00031951 |
| *phoU^†^* | Virulence |  |  | ↓ | -2.10055476 | 0.000926247 |
| *wcaM^†^* | Biofilm | Colanic acid | colanic acid biosynthesis protein WcaM | ↓ | -2.082177066 | 0.000325881 |
| *rtcA* | DNA Repair |  |  | ↓ | -2.02546902 | 0.002752772 |
| *yqhD* | Stress Response |  | putative alcohol dehydrogenase | ↓ | -2.006579875 | 0.003331559 |
| *asd^†^* | Virulence | Amino acid biosynthesis |  | ↓ | -1.963623115 | 0.001665955 |
| *stfH* | Virulence |  |  | ↓ | -1.962788323 | 0.001543697 |
| *sopF* | Virulence |  | phosphoinositide binding effector | ↓ | -1.942281238 | 0.004487083 |
| *ppdD^†^* | Virulence |  |  | ↓ | -1.937445903 | 0.044061464 |
| *purH^†^* | Virulence | Purine biosynthesis |  | ↓ | -1.851205349 | 0.027304455 |
| *foxA* | Virulence | Iron |  | ↓ | -1.837567775 | 0.00077459 |
| *pspF* | Stress Response | Phage shock regulator | phage shock protein F | ↓ | -1.809019453 | 0.00102876 |
| *tssE* | Virulence | SPI-6 | Type VI secretion system baseplate subunit TssE | ↓ | -1.796135044 | 0.03097712 |
| *cueO* | Virulence | Copper oxidase | multi copper oxidase also known as CuiD | ↓ | -1.75911423 | 0.00462846 |
| *envC* | Virulence |  | also called YibP | ↓ | -1.749688926 | 0.000222396 |
| *asrA* | Stress Response | Acid stress | anaerobic sulfite reduction subunit A | ↓ | -1.739879428 | 0.005743689 |
| *mutM* | DNA Repair |  |  | ↓ | -1.738656517 | 0.037193908 |
| *luxS* | Quorum sensing |  | S-Ribosylhomocysteinase (S-ribosylhomocysteine lyase) | ↓ | -1.725649814 | 0.010789755 |
| *yoaE* | Virulence |  |  | ↓ | -1.721139747 | 0.020460779 |
| *fliI* | Virulence | Flagella |  | ↓ | -1.688785649 | 0.007221734 |
| *pagP* | AMR |  | antimicrobial peptide resistance and lipid A acylation protein PagP | ↓ | -1.67944949 | 0.013277011 |
| *efp* | Virulence |  | translation elongation factor P (elongation factor P) | ↓ | -1.668076874 | 0.014512196 |
| *pgtC* | Virulence |  |  | ↓ | -1.661259172 | 0.004507892 |
| *yddG* | Efflux |  |  | ↓ | -1.653594635 | 0.008339695 |
| *fliK* | Virulence | Flagella |  | ↓ | -1.646198443 | 0.01531056 |
| *fepC* | Virulence | Iron | iron transporter FepBDGC | ↓ | -1.646196838 | 0.001532035 |
| *bssR* | Biofilm | Anti biofilm |  | ↓ | -1.643341213 | 0.018754392 |
| *trpB^†^* | Virulence | Amino acid biosynthesis |  | ↓ | -1.628794713 | 0.015901171 |
| *rstB* | Stress Response | Motility, invasion, iron | GerE family regulatory protein | ↓ | -1.594500115 | 0.01918833 |
| *arnT* | Virulence | Lipids | 4-amino-4-deoxy-L-arabinose transferase | ↓ | -1.59395672 | 0.002344758 |
| *fimH^†^* | Virulence | Adhesion |  | ↓ | -1.578443853 | 0.001457225 |
| *glmS* | Virulence | Cell wall | glucosamine-6-phosphate synthase | ↓ | -1.550972717 | 0.006214969 |
| *fre* | Virulence |  |  | ↓ | -1.520585141 | 0.027892978 |
| *sitA^†^* | Virulence | Iron | periplasmic chelated iron-binding protein YfeA | ↓ | -1.504771231 | 0.00432704 |
| *pbp4b* | AMR | Penicillin binding |  | ↓ | -1.499313087 | 0.045184798 |
| *guaB^†^* | Virulence | Purine biosynthesis |  | ↓ | -1.489097565 | 0.000192328 |
| *gsiA* | Virulence |  | ABC-type glutathione transporter | ↓ | -1.47231525 | 0.002065876 |
| *ygiD* | Biofilm |  |  | ↓ | -1.471348226 | 0.002068977 |
| *igaA* | Virulence |  |  | ↓ | -1.450652784 | 0.00080265 |
| *stdC^†^* | Virulence | Colonization | fimbrial chaperone protein | ↓ | -1.435047177 | 0.027304455 |
| *ttrC^†^* | Virulence | SPI-2 | tetrathionate reductase enzyme subunit | ↓ | -1.425354125 | 0.035949879 |
| *pgtE* | Virulence |  | Outer membrane protease E | ↓ | -1.413273299 | 0.001999251 |
| *fliP* | Virulence | Flagella |  | ↓ | -1.407363759 | 0.039691848 |
| *lysA* | Virulence |  |  | ↓ | -1.406914593 | 0.000714598 |
| *dacD* | AMR | Penicillin binding | D-alanyl-D-alanine carboxypeptidase DacD | ↓ | -1.391124087 | 0.005332039 |
| *yejF* | Virulence |  | ATPase component of this transporter | ↓ | -1.357853404 | 0.01071891 |
| *siiA* | Virulence | SPI-4 |  | ↓ | -1.317317293 | 0.044814287 |
| *sitD* | Virulence | Iron |  | ↓ | -1.316969445 | 0.018119952 |
| *queE* | Stress Response |  | temperature, nutrient changes | ↓ | -1.315196425 | 0.017946999 |
| *mnmE* | Virulence |  |  | ↓ | -1.303277369 | 0.001876725 |
| *cysJ* | Stress Response | Amino acid biosynthesis | sulfite reductase (NADPH) flavoprotein alpha component | ↓ | -1.302947541 | 0.000731279 |
| *sseF^†^* | Virulence | SPI-2 | secreted effector protein (secreted effector protein SseF) | ↓ | -1.281043769 | 0.04292978 |
| *kduI^†^* | Virulence |  |  | ↓ | -1.278382632 | 0.00312369 |
| *sdaB* | Virulence | Iron | L-serine deaminase II | ↓ | -1.259348942 | 0.016862308 |
| *lapB* | Virulence | Lipids | lipopolysaccharide assembly protein | ↓ | -1.237712524 | 0.019594865 |
| *fkpA^†^* | Stress Response |  | folding and peptidyl-prolyl isomerase A | ↓ | -1.237421529 | 0.022284504 |
| *proW* | Stress Response |  |  | ↓ | -1.215255792 | 0.013229043 |
| *wzz* | Virulence |  |  | ↓ | -1.210152136 | 0.004058364 |
| *acrB* | AMR | Efflux pump | acriflavine resistance protein B (multidrug efflux pump) | ↓ | -1.204801162 | 0.017181141 |
| *wcaI* | Biofilm | Colanic acid | colanic acid biosynthesis glycosyltransferase | ↓ | -1.196487991 | 0.03706399 |
| *parC* | AMR | Quinolone resistance | subunit of topoisomerase IV | ↓ | -1.192818065 | 0.000124884 |
| *zntB* | Heavy Metal | Zinc |  | ↓ | -1.180176693 | 0.034120889 |
| *katE* | Stress Response | Oxidative stress response |  | ↓ | -1.120000349 | 0.004060078 |
| *idnK^†^* | Virulence |  |  | ↓ | -1.101933102 | 0.024416948 |
| *fes* | Virulence | Iron | enterochelin esterase | ↓ | -1.095935374 | 0.03981917 |
| *tatD* | DNA Repair |  | deoxyribonuclease TatD (TatD DNase family protein) | ↓ | -1.094997673 | 0.03275329 |
| *iroC* | Virulence | Iron |  | ↓ | -1.089816635 | 0.000324031 |
| *tolB* | Virulence | Membrane | Tol-Pal system beta propeller repeat protein | ↓ | -1.089430993 | 0.041195605 |
| *hcp^†^* | Virulence | SPI-6 | putative cytoplasmic protein (type VI secretion system secreted protein Hcp) | ↓ | -1.086047412 | 0.019109458 |
| *parE* | AMR | Quinolone resistance |  | ↓ | -1.075676077 | 0.001135499 |
| *cysN* | Virulence | Amino acid biosynthesis |  | ↓ | -1.046454082 | 0.043401236 |
| *sufC* | Virulence | Fe-S | FeS assembly ATPase SufC (Fe-S cluster assembly ATP-binding protein) | ↑ | 1.003468199 | 0.019673951 |
| *seqA* | Biofilm |  | protein | ↑ | 1.01307411 | 0.009362959 |
| *elaB* | Stress Response | Membrane | Tail-Anchored Inner Membrane Protein ElaB | ↑ | 1.0189011 | 0.012307227 |
| *mdtH* | AMR | Efflux pump | multidrug resistance protein MdtH | ↑ | 1.020744279 | 0.000169825 |
| *adrA* | Biofilm |  |  | ↑ | 1.031691381 | 0.034262398 |
| *sdaA* | Virulence |  |  | ↑ | 1.042644171 | 0.000120884 |
| *ptsI* | Virulence |  | phosphoenolpyruvate-protein phosphotransferase | ↑ | 1.050147855 | 0.000183757 |
| *qseB* | Quorum sensing |  | response regulator of the QseBC two-component system (TCS) | ↑ | 1.054000317 | 0.005501749 |
| *dppF* | Virulence |  | dipeptide transport ATP-binding protein DppF | ↑ | 1.063027989 | 0.000510622 |
| *ssaR^†^* | Virulence | SPI-2, SCV |  | ↑ | 1.13401499 | 0.001009482 |
| *ptsG* | Virulence |  | pts system, glucose-specific iibc component | ↑ | 1.140204462 | 0.00017159 |
| *orgB^†^* | Virulence | SPI-1 | oxygen-regulated invasion protein OrgB | ↑ | 1.141534348 | 0.015559567 |
| *dmsB* | Stress Response | Oxidative stress response | dimethylsulfoxide reductase, chain B | ↑ | 1.198414075 | 0.004990491 |
| *ibpA^†^* | Heat Shock | Heat shock protein | small heat shock protein IbpA (molecular chaperone IbpA) | ↑ | 1.226431422 | 0.000188615 |
| *cadB^†^* | Stress Response | Acid stress |  | ↑ | 1.316483157 | 0.000698312 |
| *asrB* | Stress Response | Acid stress | anaerobic sulfite reduction subunit B | ↑ | 1.34664681 | 0.000775672 |
| *moaB* | Virulence | Molybdenum biosynthesis | molybdenum cofactor biosynthesis protein C | ↑ | 1.362660216 | 0.000297934 |
| *serA^†^* | Virulence | Amino acid biosynthesis | phosphoglycerate dehydrogenase (D-3-phosphoglycerate dehydrogenase) | ↑ | 1.376822377 | 0.002660199 |
| *cpxR^†^* | Stress Response | Stress response, biofilm | transcriptional regulatory protein CpxR | ↑ | 1.423215919 | 0.000549755 |
| *fliE* | Virulence | Flagella |  | ↑ | 1.511134685 | 0.004238692 |
| *wzb* | Biofilm | Colanic acid | low molecular weight protein-tyrosine-phosphatase wzb | ↑ | 1.715312112 | 0.003712884 |
| *fliL* | Virulence | Flagella |  | ↑ | 2.086277685 | 0.000371858 |
| *rcsA* | Virulence |  | colanic acid capsular biosynthesis activation protein A | ↑ | 2.110325939 | 0.000145625 |
| *ssrA^†^* | Virulence | SPI-2 | SPI-2 | ↑ | 9.772715988 | 0 |

^†^ Indicates that this gene is considered a virulence factor according to the FDA (https://virulence.fda.gov/data/cds/?type=g).

**Table S1C. DEG among pairwise comparison 3: Typhimurium versus monophasic Typhimurium, where monophasic Typhimurium was the reference category.**

| Gene | Category | Description | Up or Down-regulated | log2FoldChange | *p_adj_* |
| --- | --- | --- | --- | --- | --- |
| *tetR(B)* | AMR | Tetracycline resistance element repressor | ↓ | -11.51102479 | 0.002193925 |
| *ymdB* | Stress response |  | ↓ | -1.849791441 | 0.007185764 |
| *aroA*^†^ | Virulence |  | ↓ | -1.551993442 | 0.004973219 |
| *yeiP* | Virulence |  | ↑ | 1.918179113 | 0.005430709 |
| *fljA*^†^ | Flagella (repressor) |  | ↑ | 3.466280361 | 0.007137312 |
| *pepN* | Virulence |  | ↑ | 3.683786052 | 0.001617812 |

^†^ Indicates that this gene is considered a virulence factor according to the FDA (https://virulence.fda.gov/data/cds/?type=g).
