# Supplementary material for: Comparative transcriptomics of outbreak-associated Salmonella Heidelberg, Typhimurium, and monophasic Typhimurium reveals differential expression of stress tolerance, virulence, and biofilm-associated genes
Source: Front Microbiol. 2026 Oct 12;17:1895105. doi: 10.3389/fmicb.2026.1895105 (PMC13696503; doi:10.3389/fmicb.2026.1895105)
Supplement: Supplementary file 3 [file Table_2.docx]

**Table S2.** Pathway enrichment analysis using PANTHER classification for gene ontology (GO) terms reveals pathways implicated in differentially expressed genes among **(A)** monophasic *S.* Typhimurium isolates and **(B)** *S.* Typhimurium isolates compared to *S.* Heidelberg. There were no statistically significant expression profiles associated with pathways between monophasic *S.* Typhimurium and *S.* Typhimurium isolates.

**(A)**

| **Gene Ontology (GO) biological process** | **Expression (over/under)** | **Fold Enrichment** | **P-value** | **False Discovery Rate (FDR)** |
| --- | --- | --- | --- | --- |
| Cellular process (GO:0009987) | + | 1.23 | 1.41E-06 | 1.09E-03 |
| Biological process (GO:0008150) | + | 1.22 | 3.94E-08 | 9.13E-05 |

**(B)**

| **Gene Ontology (GO) biological process** | **Expression (over/under)** | **Fold Enrichment** | **P-value** | **False Discovery Rate (FDR)** |
| --- | --- | --- | --- | --- |
| Protoporphyrinogen IX biosynthetic process (GO:0006782) | + | 7.17 | 2.63E-04 | 6.08E-02 |
| Protoporphyrinogen IX metabolic process (GO:0046501) | + | 7.17 | 2.63E-04 | 5.53E-02 |
| Menaquinone biosynthetic process (GO:0009234) | + | 7.17 | 2.63E-04 | 5.07E-02 |
| Menaquinone metabolic process (GO:0009233) | + | 7.17 | 2.63E-04 | 4.68E-02 |
| Alcohol metabolic process (GO:0006066) | + | 2.85 | 5.77E-05 | 1.48E-02 |
| Organic hydroxy compound metabolic process (GO:1901615) | + | 2.69 | 2.07E-05 | 6.00E-03 |
| Small molecule metabolic process (GO:0044281) | + | 1.62 | 4.99E-07 | 1.65E-04 |
| Biosynthetic process (GO:0009058) | + | 1.61 | 3.76E-09 | 1.74E-06 |
| Metabolic process (GO:0008152) | + | 1.49 | 1.04E-13 | 5.99E-11 |
| Primary metabolic process (GO:0044238) | + | 1.42 | 1.54E-07 | 5.95E-05 |
| Cellular process (GO:0009987) | + | 1.34 | 1.64E-16 | 3.80E-13 |
| Biological process (GO:0008150) | + | 1.28 | 2.43E-15 | 1.87E-12 |
